# Psychometric properties of patient-reported outcome measures in chronic pain conditions with central sensitization- a systematic review and meta-analysis

**DOI:** 10.1186/s41687-025-00919-9

**Published:** 2025-07-11

**Authors:** Mst Farjana Akhter, Pavlos Bobos, Edith G. Otalike, Nana A. Tiwaa-Boateng, Andrew D. Firth, Igor Karp, Joel J. Gagnier

**Affiliations:** 1 Department of Epidemiology and Biostatistics, Schulich School of Medicine & Dentistry, Western University, London, ON Canada; 2 Department of Surgery, Schulich School of Medicine & Dentistry, Western University, London, ON Canada; 3 School of Physical Therapy, Western Health Science, Western University, London, ON Canada

**Keywords:** Central sensitization, Patient-reported outcome measures, Chronic pain, Measurement properties, Systematic review, COSMIN

## Abstract

**Purpose:**

To identify available patient-reported outcome measures (PROMs) used to evaluate central sensitization (CS) manifestations in chronic pain conditions and evaluate the quality of psychometric properties of those instruments.

**Methods:**

A comprehensive search across multiple electronic databases was conducted for relevant studies following the specification of eligibility criteria and development of key search terms. After screening and full-text review, the methodological quality of studies and psychometric properties of PROMs were assessed and summarized using the COnsensus-based Standards for the selection of health Measurement INstruments (COSMIN) checklist and scoring manual. The results were statistically pooled in a meta-analysis, specifically test-retest reliability, based on data availability and consistency of findings across studies.

**Results:**

A total of fifty-eight studies evaluating eight instruments in adult patients with chronic pain were included. The methodological quality of the included studies was varied. Most identified PROMs have limited evidence regarding their measurement properties. The Central Sensitization Inventory (CSI) received the highest overall ratings for most measurement properties among all the instruments, followed by Pain Sensitivity Questionnaire (PSQ) and Fibromyalgia Survey Questionnaire (FSQ). Based on pooled data from available studies, the test-retest reliability of the CSI was found to be excellent, with an intra-class correlation coefficient (ICC) of 0.93 (95% CI: 0.91–0.95) for overall chronic pain, 0.90 (95% CI: 0.87–0.93) for chronic musculoskeletal pain and 0.93 (95% CI: 0.88–0.99) for chronic neck pain. PSQ also demonstrated excellent test-retest reliability, showing an ICC of 0.86 (95% CI: 0.72–0.99) for chronic pain.

**Conclusion:**

Although not all properties have been studied, the CSI, which received the highest overall ratings, could serve as a reliable PROM assessing CS in chronic pain. More studies should be performed to comprehensively evaluate all measurement properties of all included instruments.

**Supplementary Information:**

The online version contains supplementary material available at 10.1186/s41687-025-00919-9.

## Abstract (plain language summary)

Central sensitization (CS) is a key process in many chronic pain conditions where the central nervous system becomes overly sensitive, causing persistent pain even in the absence of clear tissue damage. To evaluate the outcome of care for pain associated with CS, healthcare providers use patient-reported outcome measures (PROMs) as outcome measurement instruments that capture the patient’s pain experience. However, the quality of these PROMs is very important to ensure they accurately reflect the patient’s experience and the outcomes they intend to measure. In this study, we evaluated and rated the psychometric properties (also referred to as measurement properties) - reliability, validity and responsiveness of eight different PROMs used to assess CS manifestation in chronic pain. Our findings revealed that most PROMs lack sufficient evidence supporting their quality. Notably, the Central Sensitization Inventory (CSI) showed promising results, but further research is needed to assess all properties. Our findings recommend more studies to fully evaluate all properties of all PROMs, which would be beneficial for both clinicians and researchers to better understand, measure the outcome of care and manage chronic pain associated with CS.

**Supplementary Information:**

The online version contains supplementary material available at 10.1186/s41687-025-00919-9.

## Introduction

Chronic pain is a prevalent public health issue [1, 2]. Chronic pain adversely affects an individual’s overall quality of life, including physical and emotional well-being, sleep quality, and functional status [3, 4] and can lead to significant psychosocial consequences (e.g., depression, anxiety, social isolation, reduced work productivity) [5]. According to some studies, chronic pain causes a significant socioeconomic burden, impacting over 30% of the global population [6]. Many chronic pain patients do not have identifiable pathology for persistent pain or may continue experiencing pain even after tissue healing [7]. Central sensitization (CS) is a proposed underlying pathophysiological mechanism in chronic pain, marked by central nervous system dysregulation, neuronal imbalance, and hyperexcitability [8]. This results in heightened pain sensitivity or persistent pain despite injury resolution [9, 10]. CS is commonly associated with poor treatment response in individuals with chronic pain conditions [11–16], highlighting the importance of appropriate assessment strategies.

Patient-reported outcome measures (PROMs) are often used to measure pain associated with CS [17]. As pain is innately subjective, PROMs remain widely accepted as the standard for the assessment of an outcome of care associated with pain [18]. PROMs aim to directly capture the patient’s perspectives and experiences regarding treatment outcomes [19], which is essential for providing high-quality clinical care.

The ability of a PROM to accurately assess treatment outcomes relies on the quality of its measurement properties— reliability, validity, and responsiveness [20] because it is important to ensure that a PROM accurately measures what it intends to measure, produces consistent results in repeated administration under similar conditions and is sensitive enough to detect meaningful changes in the patient’s condition [20–22]. Furthermore, highlighting the quality of PROMs is crucial to confirm their clinical utility [23]. Several studies report that PROMs of poor quality can bias the treatment effects [e.g [24]. Therefore, data collected using measures may be unreliable or inaccurate as they may underestimate or overestimate the true effects of interventions or treatments, leading to flawed assessments which further mislead decision-making. The methodological quality of the studies focusing on the measurement properties of PROM is also crucial as it directly influences the trustworthiness of study results [25].

Therefore, assessment of the quality of measurement properties of PROMs targeting CS in chronic pain conditions is fundamental in clinical practice to derive meaningful benefit from their application. There has yet to be a systematic review conducted to evaluate the quality of all available outcome instruments used to assess CS manifestation in chronic pain with established criteria to ensure whether they possess robust measurement properties in the target population. This systematic review aims to address this research gap. The objectives of this study are to (1) identify all PROMs available to assess centralized pain or central sensitization manifestations in chronic pain conditions; (2) appraise the methodological quality of studies on measurement properties of those PROMs; (3) evaluate and summarize the quality of measurement properties of those PROMs based on their psychometric evidence and grading of the level of evidence for each measurement property.

## Methods and materials

**Fig. 1:**
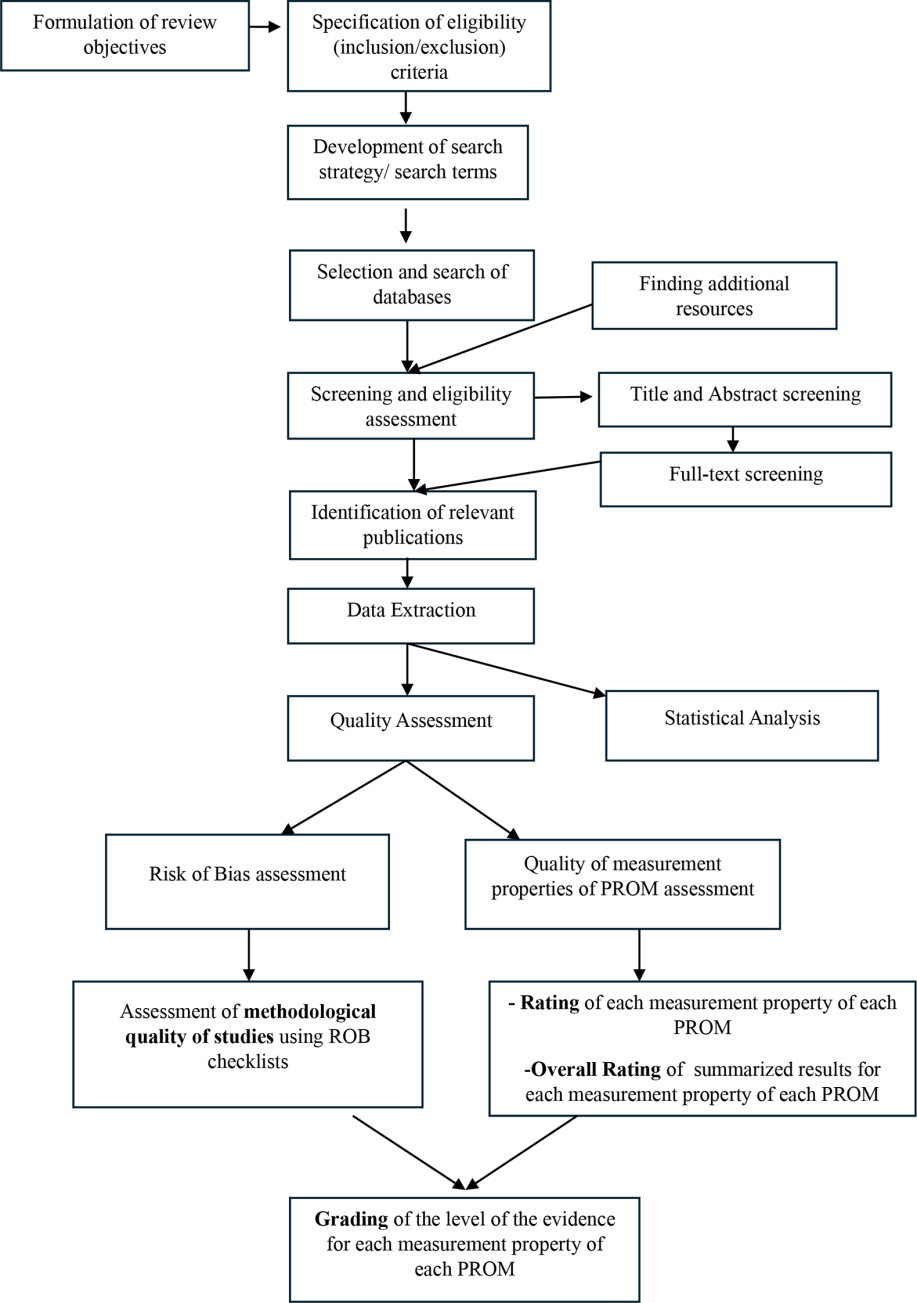
Flow diagram outlining the methodological steps

The review protocol was registered in the International Prospective Register of Systematic Reviews (CRD42023460050). This systematic review was conducted according to the COnsensus-based Standards for the selection of health Measurement INstruments (COSMIN) guidelines. Reporting adhered to the Preferred Reporting Items for Systematic Reviews and Meta-Analyses (PRISMA)-COSMIN guidelines [26]. Figure 1 presents a flow diagram summarizing the methodological steps.

### Search strategy

The following six electronic databases were searched from inception until September 2023: *MEDLINE (Ovid)*,* EMBASE*,* SCOPUS*,* PubMed*,* Web of Science*,* and CINAHL*, for studies evaluating measurement properties of PROMs assessing aspects of CS in chronic pain conditions. The search strategy was developed in collaboration with a librarian scientist and reviewed by systematic review experts to maximize sensitivity, and a parallel approach was used in the search. A search filter recommended by the COSMIN initiative for measurement properties was applied to each database [27]. Additional searches were conducted by combining the terms related to CS, chronic pain condition and PROMs. The full search strategy is available (Appendix A). The reference lists of identified articles were also manually screened to find more relevant studies.

### Eligibility criteria and study screening

Two reviewers (FA and NT) independently assessed studies for eligibility. Covidence software (Covidence systematic review software, Veritas Health Innovation, Melbourne, Australia, available at www.covidence.org) was used to facilitate the organization of records retrieved from searches. This process enabled the detection and elimination of any instances of duplication. Studies were included if they met the following criteria: (1) full-text articles published in English; (2) Studies that evaluated CS manifestations using PROMs in adult patients with chronic pain conditions; (3) Studies that evaluated measurement properties of either an original or translated version of PROM; and/or (4) Studies that reported the development and/or assessment of the interpretability of the PROMs. Studies were excluded if they: (1) included populations that were not representative of adults with chronic pain experiencing central sensitization; (2) were not designed to evaluate the measurement properties of PROM; (3) assessed instruments not intended to capture manifestations of CS in chronic pain conditions; (4) utilized the PROM solely as part of the validation process for another instrument.

Initially, the titles and abstracts were screened based on the eligibility criteria. The full-text screening was then performed on citations. A third reviewer (AF) resolved the disagreement regarding the disputed articles.

### Data extraction

Two reviewers (FA, EO) independently extracted data from the included studies. The following information was extracted: (1) characteristics of included studies (author/year, country, name of PROMs, objectives of the study, sample size, mean age, sex, and chronic pain types, (2) characteristics of PROMs (name of the instrument, response option, scoring system, and the original language of the instruments).

### Quality assessment

Quality assessment was conducted by using the COSMIN checklist and scoring manual based on nine measurement properties [22, 25]. In this review, the quality assessment of the included PROMs involved three stages. Two reviewers (FA, EO) independently conducted the quality assessments. In cases where a consensus couldn’t be reached, reviewer conflicts were referred to an expert researcher (JG), who helped in reaching a consensus.

### Stage 1. COSMIN risk of Bias checklist

Evaluation of the methodological quality of each study was performed using the COSMIN risk of bias checklist [22, 28]. There are 4 to 13 items in each box of the checklist for each measurement property which are rated using a four-point rating scale and the overall rating relies on the lowest favourable rating (which is called “worst score counts”) given to any of the items within each measurement property [25, 28].

### Stage 2. Employing updated criteria for good measurement properties

#### Stage 2a: content validity

Each study’s findings on PROM development and content validity were evaluated using ten criteria for content validity [29]. The evaluations of all available studies were then qualitatively summarized to determine whether the relevance, comprehensiveness, comprehensibility, and overall content validity were sufficient (+), insufficient (-), indeterminate (?), or inconsistent (±) considering all evidence [29].

#### Stage 2b: remaining measurement properties

For instruments with sufficient content validity, each study’s results on the remaining measurement properties were evaluated using updated criteria for good measurement properties (Table 1) and rated as sufficient (+), insufficient (-), or indeterminate (?) [25].

Although updated COSMIN criteria provide fit indices for confirmatory factor analysis, they do not specify a cutoff for acceptable factor loading in exploratory factor analysis (EFA). In this review, a 0.4 cutoff was used for EFA based on existing literature [30]. Test-retest reliability measures the stability of test results over time by administering the same test to the same group of people on different occasions. The time interval between test-retest is an important factor for evaluating reliability, and a period of around two weeks is often regarded as a suitable interval between two administrations. However, it depends on the specific construct being assessed and the underlying condition [21, 22]. In this review, a timeframe ranging from 5 days to 2 weeks was considered appropriate. Convergent validity was assessed using COSMIN’s recommended generic hypotheses, considering both the expected direction and magnitude of correlations between the PROM and comparator instruments [22, 25]. It is important to consider clearly defined constructs and adequate measurement properties of the comparator instrument(s) within a population similar to the study population [22]. Discriminative validity was assessed by expecting a 10-point difference in PROM scores between patient subgroups and pain-free controls. This property is critical in determining a PROM’s ability to differentiate between groups with distinct characteristics, as highlighted in the COSMIN methodology for systematic reviews [22].

### Stage 3. Summary of evidence

#### Stage 3a. Content validity

Each PROM’s overall ratings of content validity were determined, followed by grading the level of evidence using a modified GRADE approach (Tables 2 and 3) by considering four factors (risk of bias, inconsistency, imprecision, and indirectness) [25].

#### Stage 3b. Remaining measurement properties

The findings of available studies were summarized qualitatively and rated again using the updated COSMIN criteria to conclude whether, overall, the remaining measurement properties (internal consistency, reliability, measurement error, structural validity, hypothesis testing for construct validity, cross-cultural validity, criterion validity and responsiveness) of each PROM are sufficient (+), insufficient (−), inconsistent (±), or indeterminate (?). As a general guideline, a minimum of 75% of the results should align with the criteria to rate the qualitatively summarized results as sufficient (or insufficient) [22, 25]. The quality of the evidence of each measurement property was then graded using the modified GRADE approach.

### Statistical analysis

In this review, the reliability coefficients (intraclass correlation coefficient) of available studies were statistically pooled in a meta-analysis. The meta-analysis of the reliability coefficient was conducted by fitting a random-effects model specified with the restricted maximum likelihood method using Stata. To facilitate the interpretation of the meta-analysis results, the average reliability coefficients and their confidence limits, obtained through Fisher’s Z transformations, were converted back to intraclass correlation metrics. ‘Do file’ outlining codes for meta-analysis is available (Appendix B). Heterogeneity was evaluated using the Q statistic and the I² index and visualized through a forest plot.

**Table 1:** Updated criteria for good measurement properties

Measurement properties	Definition	Rating	Quality Criteria**
Reliability			
*Internal consistency*	The degree of interrelatedness among the items	+	At least low evidence for sufficient structural validity AND Cronbach’s alpha(s) ≥0.70 for each unidimensional scale or subscale Criteria for “At least low evidence for sufficient structural validity” not met At least low evidence for sufficient structural validity AND Cronbach’s alpha(s) < 0.70 for each unidimensional scale or subscale
		?	
		-	
*Reliability*	The proportion of the total variance in the measurements which is due to “true” differences between patients	+	ICC or weighted Kappa ≥ 0.70 ICC or weighted Kappa not reported ICC or weighted Kappa < 0.70
		?	
		-	
*Measurement error*	The systematic and random error of a patient’s score that is not attributed to true changes in the construct to be measured	+	SDC or LoA < MIC MIC not defined SDC or LoA > MIC
		?	
		-	
**Validity**			
*Content validity*	The degree to which the content of an instrument is an adequate reflection of the construct to be measured	+	Ten criteria for good content validity [29]
		?	
		-	
*Structural validity*	The degree to which the scores of a PROM are an adequate reflection of the dimensionality of the construct to be measured	+	CTT: CFA: CFI or TLI or comparable measure > 0.95 OR RMSEA < 0.06 OR SRMR < 0.08 IRT/Rasch: No violation of unidimensionality: CFI or TLI or comparable measure > 0.95 OR RMSEA < 0.06 OR SRMR < 0.08 AND no violation of local independence: residual correlations among the items after controlling for the dominant factor < 0.20 OR Q3's < 0.37 AND no violation of monotonicity: adequate looking graphs OR item scalability > 0.30 AND adequate model fit: IRT: χ2 > 0.01 Rasch: infit and outfit mean squares ≥ 0.5 and ≤ 1.5 OR Z- standardized values > − 2 and < 2 CTT: Not all information for ‘+’ reported IRT/Rasch: Model fit not reported Criteria for ‘+’ not met
		?	
		-	
Validity			
*Hypotheses testing for construct validity*	The degree to which the scores of a PROM are consistent with the hypothesis (for instance with regard to internal relationships, relationships to scores of other instruments, or differences between relevant groups) based on the assumption that the PROM validly measures the construct to be measured	+	The result is in accordance with the hypothesis No hypothesis defined (by the review team) The result is not in accordance with the hypothesis
		?	
		-	
*Cross-cultural validity\measurement invariance*	The degree to which the performance of the items on a translated or culturally adapted PROM is an adequate reflection of the original version of the PROM	+	No important differences were found between group factors (such as age, gender, language) in multiple group factor analysis OR no important DIF for group factors (McFadden's R² < 0.02) No multiple group factor analysis OR DIF analysis performed Important differences between group factors OR DIF was found
		?	
		-	
*Criterion validity*	The degree to which the scores of a PROM are an adequate reflection of a “gold standard	+	Correlation with gold standard ≥ 0.70 OR AUC ≥ 0.70 Not all information for ‘+’ reported Correlation with gold standard < 0.70 OR AUC < 0.70
		?	
		-	
**Responsiveness**			
*Responsiveness*	The degree to which the scores of a PROM to detect change over time in the construct is to be measured	+	The result is in accordance with the hypothesis OR AUC ≥ 0.70 No hypothesis defined (by the review team) The result is not in accordance with the hypothesis OR AUC < 0.70
		?	
		-	

Quality criteria** was adapted from the COSMIN guidelines for systematic reviews of PROMs [22, 25], “+” = sufficient, “−” = insufficient, “?” = indeterminate, AUC = area under the curve, CFA = confirmatory factor analysis, CFI = comparative fit index, CTT = classical test theory, DIF = differential item functioning, ICC = intraclass correlation coefficient, IRT = item response theory, LoA = limits of agreement, MIC = minimal important change, RMSEA: root mean square error of approximation, SEM = standard error of measurement, SDC = smallest detectable change, SRMR: standardized root mean residuals, TLI = Tucker–Lewis index

**Table 2:** Modified GRADE approach*

Quality of evidence	Lower if
High	**Risk of bias**
Moderate	-1 Serious
Low	-2 Very serious
Very low	-3 Extremely serious
	**Inconsistency** -1 Serious -2 Very serious
	**Imprecision** -1 total *n* = 50–100 -2 total *n* < 50
	**Indirectness** -1 Serious -2 Very serious

Modified GRADE approach* was adapted from the COSMIN guidelines for systematic reviews of PROMs [22, 25]

**Table 3:** Guidance on reducing grade for risk of bias

Risk of bias	Downgrading for Risk of Bias*
No	There are multiple studies of at least adequate quality, or there is one study of very good quality available
Serious	There are multiple studies of doubtful quality available, or there is only one study of adequate quality
Very serious	There are multiple studies of inadequate quality, or there is only one study of doubtful quality available
Extremely serious	There is only one study of inadequate quality available

Downgrading for Risk of Bias* was adapted from the COSMIN guidelines for systematic reviews of PROMs [22, 25]

## Results

### Search results

**Fig. 2:**
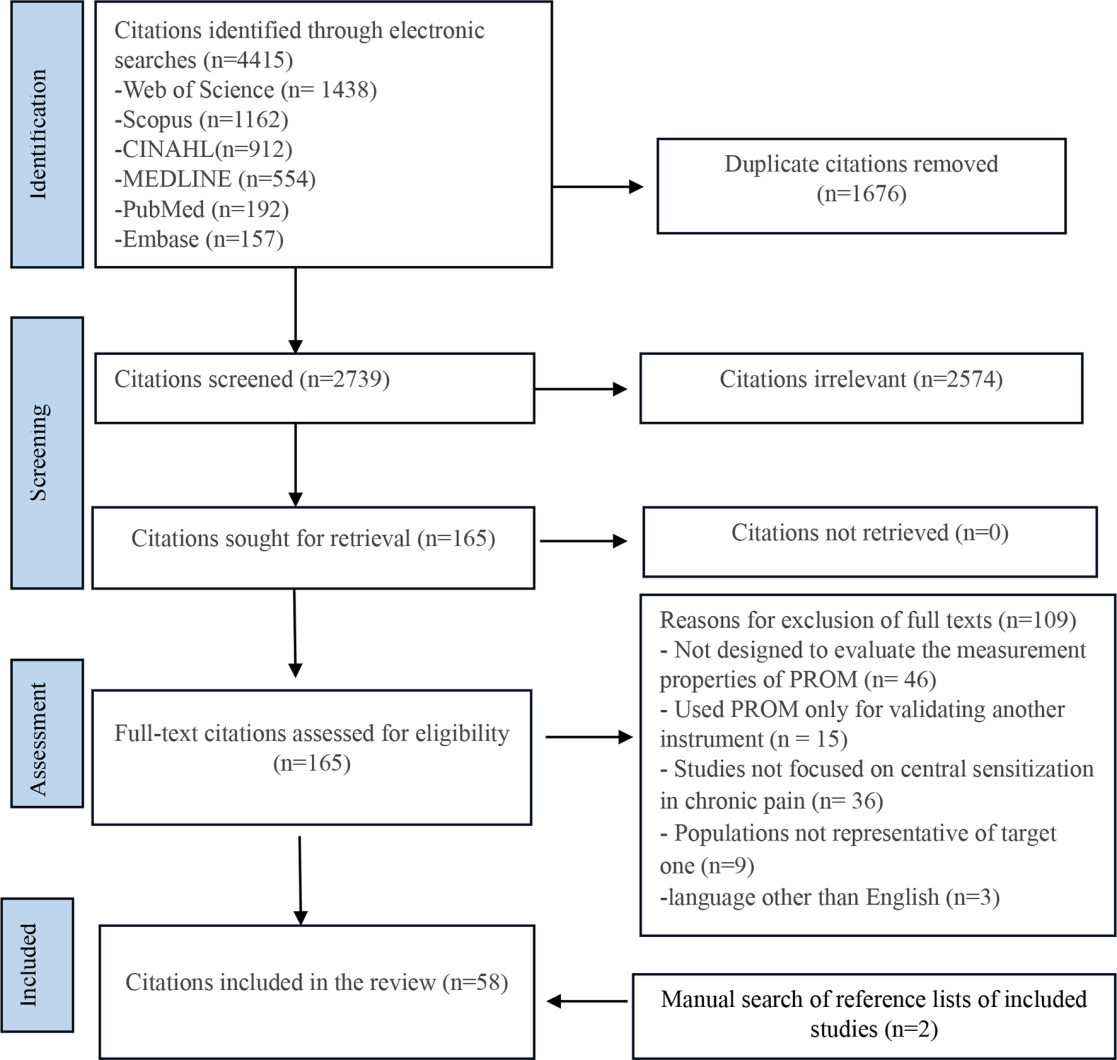
PRISMA flowchart outlining the process of selecting studies

The database search identified fifty-eight eligible studies for this review (Fig. 2). Sixteen studies investigated the measurement properties of the original PROMs, and forty-two studies assessed the translated version. The number of participants in the studies ranged from 31 [31] to 1651 [75] with a mean patient age of 48 years (SD = 11.8). The distribution of male and female participants in the studies varied, with some studies including only female participants [58, 72, 80, 82, 83] and the remaining studies having a disproportionate ratio of females to males. The characteristics of the fifty-eight included studies [30–87] are presented in Table 4. The included studies covered various chronic pain conditions (e.g., fibromyalgia, chronic low back pain, neck pain, Chronic widespread pain, musculoskeletal pain, mixed chronic pain) and evaluated eight different PROMs, with the number of items ranging from seven to twenty-five and subscales/domains from two to nine (Table 5). The most frequently assessed instruments were the Central Sensitization Inventory (CSI) (thirty-seven studies), the Fibromyalgia Survey Questionnaire (FSQ) (nine studies), and the Pain Sensitivity Questionnaire (PSQ) (eight studies). The other instruments, each of which measurement properties were assessed in a single study. Notably, Coronado and George (2018) concurrently evaluated the CSI and PSQ, leading to separate reporting and rating of these instruments’ psychometric properties in this review.

### COSMIN quality assessment results

Table 6 (Appendix C) provides COSMIN ratings on methodological quality, results and overall rating per measurement property. A summary of COSMIN overall ratings of each measurement property of each PROM is provided in Table 7, while Table 8 illustrates the grading of the quality of evidence for measurement properties of PROMs.

The central sensitization Inventory (CSI) was rated ‘sufficient’ for content validity, structural validity, internal consistency, reliability, hypothesis testing for construct validity (83% of the results aligned with the hypotheses) and responsiveness. However, CSI was rated ‘indeterminate’ for measurement error because some studies assessed measurement error, but they failed to calculate the minimal important change (MIC) value. CSI received a ‘high’ quality of evidence score for most of the reported measurement properties (internal consistency, reliability, construct validity and responsiveness) except structural validity and content validity. Due to a lack of clear reporting regarding the assessment of the content validation process, the quality of the studies received a “doubtful’ rating. Therefore, the level of evidence for content validity of CSI was downgraded to ‘moderate’ for risk of bias. Again, the results of available studies on structural validity were rated as ‘sufficient’ (60%) and ‘insufficient’ (40%). Therefore, the level of evidence was downgraded to ‘moderate’ for inconsistency. Furthermore, the quality of evidence for measurement error was not graded due to the absence of MIC value [30–66].

The Pain Sensitivity Questionnaire (PSQ) was rated ‘sufficient’ for content validity, structural validity, internal consistency, and reliability. However, PSQ was rated ‘insufficient’ for construct validity (> 25% of study results were not aligned with the hypotheses). The quality of evidence of PSQ was scored ‘high’ for internal consistency and construct validity but ‘low’ for structural validity and reliability, and ‘moderate’ for content validity. The level of evidence was rated as ‘low’ because the included studies were of ‘inadequate’ methodological quality. A ‘moderate’ level of evidence for content validity was found due to inappropriate descriptions of the content validation process in included studies [46, 67–73].

The Fibromyalgia Survey Questionnaire (FSQ) was rated ‘sufficient’ for content validity, reliability, and construct validity. However, internal consistency for FSQ was rated ‘indeterminate’ because of the absence of factor analysis to provide evidence for unidimensional factor structure, resulting in a downgrade of the COSMIN rating. FSQ received a ‘high’ quality of evidence score for internal consistency and construct validity. A ‘moderate evidence was found for content validity and reliability due to insufficient descriptions of the content validation process and the use of inappropriate statistical methods, respectively, leading to predominantly “doubtful” methodological ratings. Therefore, the level of evidence for these properties was downgraded to ‘moderate’ for risk of bias [74–82].

The Nociplastic-based Fibromyalgia Features (NFF) tool was rated ‘sufficient’ for criterion validity and construct validity. Again, the internal consistency of NFF received an ‘indeterminate’ rating due to the absence of factor analysis. NFF quality of evidence was scored ‘high’ for these three measurement properties [83].

The Generalized Pain Questionnaire (GPQ) was rated ‘sufficient’ for structural validity, internal consistency, and construct validity. However, assessments of other measurement properties were absent. GPQ received a ‘high’ quality of evidence score for internal consistency, ‘moderate’ for construct validity, but ‘very low’ for structural validity (because only one methodologically ‘inadequate’ study due to insufficient sample size) [84].

The Sensory Hypersensitivity Scale (SHS) was rated ‘insufficient’ for construct validity because 60% of the study results didn’t correspond with the hypotheses. However, due to the absence of evidence for sufficient structural validity, SHS was rated ‘indeterminate’ for internal consistency. SHS quality of evidence was scored ‘high’ for internal consistency, and ‘moderate’ for construct validity because only one methodologically ‘adequate’ study [85].

The Novel Self-report Questionnaire was rated ‘sufficient’ for structural validity, internal consistency, and construct validity. This tool received ‘low’ for the quality of evidence of structural validity and construct validity because of the insufficient sample size (˂100) and only one study with ‘doubtful’ quality, respectively. However, this questionnaire was rated ‘high’ for its internal consistency [86].

The Leiden Visual Sensitivity Scale (L-VISS) & Visual Discomfort Scale (VDS) were rated ‘sufficient’ for construct validity. Internal consistency was rated ‘indeterminate’, because of the absence of structural validity assessment. The quality of evidence was scored ‘High’ for internal consistency. However, ‘low’ evidence was found for construct validity due to only one study with ‘doubtful’ quality [87].

A few studies reported missing data in their assessments of structural validity, and information on floor and ceiling effects was available in only a limited number of studies. Further details and interpretations of these findings are provided in the Discussion section.

**Table 4:** Characteristics of included studies

Author (ref)	Country	PROM	Objective of the study	Sample size (*N*)	Age mean ± SD (range) year	Sex (% Female)	Chronic pain	
							Type	Conditions
Mayer et al., 2012 [30]	USA	CSI	To develop the Central Sensitization Inventory (CSI) and to assess the psychometric validity and clinical utility of the CSI to differentiate among different subject groups of chronic pain patients.	Study 1: 149 participants for Reliability and 359 participants including both chronic pain patients (210 patients) and healthy participants for factor analysis Study 2: 105 chronic pain patients and 40 healthy participants	Study 1: 22 ± 4.7 Study 2: FM = 47 ± 7.6, CWP = 46 ± 11.5, CLBP = 43 ± 10.0, Control group= 21 ± 13.6	Study1: 70 Study 2: FM= 73, CWP = 26, CLBP = 25, Control group = 77	Mixed chronic pain	Fibromyalgia (FM *n* = 30), chronic widespread pain without FM (CWP *n* = 31), chronic low back pain (CLBP *n* = 44)
Bid et al., 2016 [31]	India	CSI	To translate and cross-culturally adapt the CSI into Gujarati, and subsequently check its psychometric properties among patients with Chronic low back pain.	20 patients for content validity, 31 patients for other psychometric evaluation	53 ± 13.2	23 (74.2)	Chronic musculoskeletal pain	Chronic low back pain (CLBP)
Noorollahzadeh et al., 2021 [32]	Iran	CSI	To perform translation, cultural adaptation of CSI into Persian and to assess its psychometric properties.	20 patients for pretesting, and 256 patients for validation studies	patient group = 42 ± 11.8, Control group = = 36 ± 9.9	98 (38.3)	Mixed chronic pain	Chronic regional pain syndrome, restless leg syndrome, hip, knee and spinal (cervical, thoracic and lumbar), osteoarthritis, spondylolisthesis, frozen shoulder, coccydynia, trigeminal neuralgia
Liang et al., 2022 [33]	China	CSI	To adapt CSI to Chinese and test its validity and reliability	6 patients for pilot testing, 237 patients for validation studies	44 ± 12.7	223 (76.4)	Mixed chronic pain	Fibromyalgia (FM *n* = 114), musculoskeletal pain (*n* = 123: Hip pain, knee pain, ankle pain, shoulder pain, elbow pain, hand and wrist pain, lateral epicondylitis and temporomandibular joint pain, lumbago, back pain, cervicodynia)
Roby et al., 2022 [34]	Canada	CSI	To evaluate the validity of the CSI through Rasch analysis in knee osteoarthritis patients	293 patients for validation studies	64 ± 9.5	172 (58.7)	Chronic musculoskeletal pain	Knee osteoarthritis
Bakhtadze et al., 2022 [35]	Russia	CSI	To investigate the psychometric properties of the Russian version of the CSI in patients with non-specific neck pain associated with migraine and tension-type headache.	204 patients	38 ± 10.5	173 (84.8)	Mixed chronic pain	Non-specific neck pain(*n* = 37), non-specific neck + episodic headache (*n* = 30), non-specific neck + chronic headache (*n* = 137)
Wiangkham et al., 2022 [36]	Thailand	CSI	To translate and cross-culturally adapt the CSI into Thai, and subsequently evaluate its psychometric properties among patients with chronic non-specific neck pain.	30 patients for the cross-cultural adaptation process, and 340 patients for psychometric evaluation	34 ± 14 (20–68)	226 (66.50)	Chronic musculoskeletal pain	Chronic non-specific neck pain
Hendriks et al., 2020 [37]	Netherland	CSI	To evaluate the convergent validity of the Dutch version of the CSI in chronic whiplash-associated patients.	125 patients	40 ± 11.3 (18–65)	71 (56.8)	Chronic musculoskeletal pain	Chronic whiplash-associated disorder
Bakhtadze et al., 2021 [38]	Russia	CSI	To evaluate the validity and reliability of the Russian version of the CSI in patients with chronic non-specific neck and back pain.	195 patients for validation studies	41 ± 11.4 (18–65)	142 (72.8)	Chronic musculoskeletal pain	Chronic non-specific neck pain (*n* = 106) and /or back pain (*n* = 75)
Sharma et al., 2020 [39]	Nepal	CSI	To translate and culturally adapt the CSI into Nepali and to test its internal consistency, test-retest reliability, measurement error and construct validity among patients with subacute and chronic musculoskeletal pain.	20 patients for pretesting and 115 patients for psychometric evaluation	42 ± 14.6	67 (58)	Chronic musculoskeletal pain	Not reported
Düzce Keleş et al., 2021 [40]	Turkey	CSI	To translate the CSI into Turkish and perform a psychometric validation among patients with chronic spinal pain of organic origin and fibromyalgia patients.	200 patients and 100 controls for psychometric evaluation	Fibromyalgia = 45 ± 8.4 (25–60), CSPO = 44 ± 9.7 (21–60), Healthy control = 36 ± 10.1 (25–55)	276 (92)	Mixed chronic pain	Fibromyalgia patients with widespread pain (FMWP *n* = 100), chronic spinal pain of organic origin (CSPO *n* = 100)
Salaffi et al., 2022 [41]	Italy	CSI	To evaluate the convergent and discriminant validity of the Italian version of the CSI in patients with fibromyalgia.	562 FM patients	53 ± 9.6	511 (90.1)	Mixed chronic pain	Fibromyalgia
Tanaka et al., 2017 [42]	Japan	CSI	To test criterion validity and construct validity of the Japanese version of the CSI and to investigate prevalence rates of CS severity levels in patients with musculoskeletal disorders.	6 patients for pretesting, 290 patients for validation studies	51 ± 15.6	188 (64.83)	Mixed chronic pain	Musculoskeletal pain disorders (neck injury (including whiplash) shoulder, low back, hip, knee, or ankle pain) restless leg syndrome, chronic fatigue syndrome, irritable bowel syndrome, temporomandibular joint disorder, migraine, or tension headaches [No CSS (*n* = 209), 1 CSS (*n* = 63), 2 and above CSSs (*n* = 18)]
Neblett et al., 2013 [43]	USA	CSI	To investigate the original version of the CSI scores in chronic pain patients with Central sensitization syndrome, and nonclinical samples to determine a clinically relevant cutoff value.	121 patients and 129 nonpatient comparison sample	CSS patients = 45 ± 13.3, non-CSS patients = 46 ± 12.2, non-patient comparison sample = 21 ± 3.6	167 (66.8)	Mixed chronic pain	Central sensitization syndrome (CSS *n* = 89: tension headaches/migraines, myofascial pain syndrome, fibromyalgia, IBS, TMD and PTSD), non-CSS (*n* = 32)
Caumo et al., 2017 [44]	Brazil	CSI	To examine the psychometric characteristics of the Brazilian-adapted CSI, including internal consistency, construct validity, reproducibility, and factor structure among patients with chronic musculoskeletal pain.	20 patients for pretesting, 222 patients and 63 nonpatients for the comparison sample	OA = 67 ± 8.2 MPS = 43 ± 11.5 CTTH = 36 ± 12.2 FM = 50 ± 11 Control group = 38 ± 14.34	248 (87)	Mixed chronic pain	Osteoarthritis (OA *n* = 31), myofascial pain syndrome (MPS *n* = 65), chronic tension-type headache (CTTH *n* = 53), fibromyalgia (FM *n* = 73),
Kregel et al., 2016 [45]	Netherland	CSI	To perform psychometric properties assessment of Dutch CSI among patients with chronic pain.	368 patients for validation studies.	Chronic pain patients = 43 ± 13.2 Healthy control = 37 ± 14.8	269 (64.5)	Chronic musculoskeletal pain	Not reported
Coronado and George, 2018 [46]	USA	CSI	To assess construct validity and concurrent validity of the original version of CSI and PSQ among patients with shoulder pain.	78 patients	Patients group = 39 ± 14.5 Control group = 35 ± 11.1	36 (46.2)	Chronic musculoskeletal pain	Shoulder pain
Feng et al., 2022 [47]	China	CSI	To create the Chinese Cultural Adaptation of the CSI and assess its psychometric properties in chronic pain patients.	30 patients for pretesting, and 235 patients for validation studies	64 ± 9.5	196 (83.40)	Chronic musculoskeletal pain	Not reported
Cuesta-Vargas et al., 2016 [48]	Spain	CSI	To translate the CSI into Spanish and subsequently conduct its psychometric validation among chronic pain patients.	395 patients	56 ± 12.7	176 (44.4)	Chronic musculoskeletal pain	Not reported
Knezevic et al., 2018 [49]	Serbia	CSI	To translate the original CSI into Serbian and to examine its psychometric properties in chronic pain patients.	8 patients for cognitive debriefing and 355 patients for validation studies	52 ± 12.9	233 (65.6)	Mixed chronic pain	Complex regional pain syndrome(*n* = 48), fibromyalgia (*n* = 23), low back pain, cervical pain, knee pain, hip pain, ankle pain, shoulder pain, lateral epicondylitis, temporomandibular joint pain
Mikkonen et al., 2021 [50]	Finland	CSI	To translate and cross-culturally adapt the CSI into Finnish (CSI-FI) and subsequently test its reliability and validity.	20 patients for face validity, 187 patients and 42 controls for validation studies	Chronic pain group: 40 ± 10.6, Control group: 46 ± 11.8	162 (70.7)	Mixed chronic pain	Chronic low back pain, other chronic musculoskeletal pain, chronic headache
Neblett et al., 2017 [51]	USA	CSI	To establish a gradient of clinically relevant symptom severity levels for the original version of CSI.	287 patients (Study-2)	Average age = 50	201 (70)	Mixed chronic pain	CSS- Fibromyalgia, tension/migraine headaches, post-traumatic Stress disorder, restlesslLeg syndrome, temporomandibular joint disorder, chronic fatigue syndrome, irritable bowel syndrome [No CSS (*n* = 120), 1 CSS (*n* = 109), 2 CSSs (*n* = 40), 3 CSSs (*n* = 6), 4 + CSSs(*n* = 12)]
Kosińska et al., 2021 [52]	Poland	CSI	To validate the Polish CSI in patients with chronic spinal pain.	151 patients and 30 healthy controls	patient group = 56 ± 14.1 controls = 42 ± 12.6	145 (80.1)	Chronic musculoskeletal pain	Chronic neck pain (CNP *n* = 24), chronic low back pain (CLBP *n* = 73), both CNP and CLBP(*n* = 54)
Bilika et al., 2020 [53]	Greece	CSI	To translate and cross-culturally adapt the CSI into Greek and to perform psychometric properties assessment among patients with chronic pain.	20 patients for pilot testing and 200 patients for validation studies	patient group = 49 ± 14.7, controls = 28 ± 8.7	149 (59.6)	Mixed chronic pain	Low back pain, cervical pain, knee pain, hip pain, ankle pain, shoulder pain, epicondylitis, temporomandibular joint pain (multiple pain complaint *n* = 55; single pain complaint *n* = 115), and fibromyalgia (*n* = 30)
Kregel et al., 2018 [54]	Belgium	CSI	To assess the convergent validity of the Dutch version of the CSI, and to explore its relationship with psychophysical pain measures and self-reported assessments in patients experiencing chronic spinal pain.	116 patients	40 ± 12.5	72 (62.1)	Chronic musculoskeletal pain	Non-specific chronic spinal pain (including CLBP *n* = 54; chronic idiopathic neck pain *n* = 62)
Kim et al., 2020 [55]	Korea	CSI	To translate the CSI into the Korean version and investigate its psychometric properties in patients with knee osteoarthritis.	20 patients for pilot testing and 269 patients for validation studies	71 ± 7.7	236 (87.7)	Chronic musculoskeletal pain	Knee osteoarthritis
van der Noord et al., 2018 [56]	Netherland	CSI	To investigate the convergent validity and to present clinically relevant categories for the Dutch CSI in chronic pain patients.	198 patients	47 ± 15.5	115 (58.1)	Chronic pain	Not reported
Chiarotto et al., 2018 [57]	Italy	CSI	To cross-culturally adapt the CSI into Italian, and subsequently evaluate its structural and construct validity in chronic pain patients.	20 patients for pilot testing and 220 patients for validation studies	55 ± 15.5	172 (78.8)	Mixed chronic pain	Low back pain (LBP *n* = 73), temporomandibular disorder (TMD *n* = 37), hand osteoarthritis (HOA *n* = 43), fibromyalgia (FM *n* = 20), or rheumatoid arthritis (RA *n* = 44).
Valera-Calero et al., 2022 [58]	Spain	CSI	To analyze the convergent validity of CSI with psychological and psychophysical outcomes in patients with Fibromyalgia.	126 patients	53 ± 11.0	126 (100)	Mixed chronic pain	Fibromyalgia (FM)
Madi et al., 2021 [59]	Jordan	CSI	To adapt CSI into Arabic and to investigate its psychometric properties in chronic pain patients.	15 patients for pre-testing and 171 patients for validation studies	37 ± 12.9	128 (74.9)	Mixed chronic pain	Not reported
Knezevic et al., 2020 [60]	Serbia	CSI	To explore evidence of convergent and discriminant validity of the Serbian version of the CSI in chronic pain patients.	399 patients and 146 control subjects	49 ± 14.5	361 (67.5)	Mixed chronic pain	Low back pain (*n* = 155, cervical pain(*n* = 26), knee pain, hip pain, ankle pain, shoulder pain, lateral epicondylitis (Pain in 2 or more locations *n* = 95), temporomandibular joint pain (TMJ *n* = 20), complex regional pain syndrome (*n* = 46), and fibromyalgia (FM *n* = 47)
Van Wilgen et al., 2018 [61]	Netherland	CSI	To examine the convergent validity of the Dutch version of the CSI in chronic pain patients	114 patients	47 ± 15.9	64 (56.1)	Chronic pain	Not reported
Klute et al., 2021 [62]	Germany	CSI	To cross-culturally adapt the CSI into German version, and subsequently evaluate its psychometric properties in chronic pain patients.	247 patients and 63 controls	55 ± 13.1	217 (70)	Mixed chronic pain	Fibromyalgia syndrome (FMS *n* = 37), multisite chronic pain (MCP *n* = 63), regional chronic pain (RCP *n* = 17), chronic back and/or neck pain (CBNP *n* = 83), rheumatoid arthritis in remission (RAR *n* = 47)
Holm et al., 2021 [63]	Denmark	CSI	To examine the convergent validity of the Danish version of the CSI with quantitative sensory testing and self-reported psycho-social questionnaires in patients with low back pain.	168 patients	56 ± 14.9	65 (39.1)	Chronic musculoskeletal pain	Low back pain
Neblett et al., 2015 [64]	USA	CSI	To determine the ability of the CSI to distinguish between chronic pain patients, with and without central sensitivity syndromes.	161 patients	44 ± 11.6	92 (57.14)	Chronic pain	Not reported
Neblett et al., 2017 [65]	USA	CSI	To assess CSI scores and their associations with other clinically relevant psychosocial variables in patients with chronic spinal pain disorder.	763 patients	47 ± 10.6	270 (35%)	Chronic musculoskeletal pain	Chronic spinal pain
Bid et al., 2017 [66]	India	CSI	To compare whether the McKenzie exercise program (MEP) reduces CS better than the Conventional physiotherapy program (CPP) in patients with chronic non-specific low back pain by using the Gujarati version of CSI.	128 patients	Patients group: 41 ± 7.3 Control group: 41 ± 7.76	Experimental: 36 (46.2) Control: 42 (53.8)	Chronic musculoskeletal pain	Chronic non-specific low back pain.
Ruscheweyh et al., 2012 [67]	Germany	PSQ	To assess the validity of the PSQ in chronic pain patients.	134 patients and 185 healthy control subjects	control = 45 ± 21; CTTH = 42 ± 17 CLBP = 49 ± 15; TMD = 48 ± 14; Mixed = 50 ± 13	195 (61.1)	Mixed chronic pain	Chronic tension-type headache, chronic low back pain (CLBP), chronic temporomandibular disorder (TMD), mixed chronic pain
Sellers et al., 2013 [68]	USA	PSQ	To validate the English version of PSQ in the chronic pain population.	136 patients	54 ± 14	83 (61.0)	Chronic musculoskeletal pain	Chronic low back pain (CLBP)
Ibancos-Losada et al., 2021 [69]	Spain	PSQ	To cross-culturally adapt the PSQ into Spanish and subsequently analyze its psychometric properties in fibromyalgia syndrome patients.	15 participants for pilot testing 58 patients and 296 controls for validation studies	37 ± 11.9	235 (66.4)	Mixed chronic pain	Fibromyalgia syndrome (FMS)
Inal et al., 2021 [70]	Turkey	PSQ	To translate the PSQ into Turkey and investigate its validity in chronic pain patients.	10 participants for pilot testing and 73 patients for validation studies	57 ± 15.4 (18–90)	48 (65.8)	Chronic musculoskeletal pain	Chronic back pain
Latka et al., 2019 [71]	Poland	PSQ	To translate and cross-culturally adapt the CSI into Polish and to perform psychometric properties assessment among patients with low back pain.	12 patients for pretesting and 144 patients for validation studies	53 (19–80)	64 (44.4)	Chronic musculoskeletal pain	Low back pain
Grundström et al., 2019 [72]	Sweden	PSQ	To assess associations between the Sweedish version of PSQ scores and QST and to determine the extent of psychological distress influenced PSQ scores.	37 patients	Patients group = 26 ± 5.9 (18–40) Control = 30 ± 5.6 (18–40)	37 (100)	Mixed chronic pain	Persistent pelvic pain
Kim et al., 2014 [73]	Korea	PSQ	To translate and cross-culturally adapt the CSI into Korean and subsequently conduct its psychometric validation among chronic pain patients.	30 patients for pretesting and 72 patients for validation studies	66 ± 8.1	45 (62.5)	Chronic musculoskeletal pain	Chronic leg pain and/or back pain caused by degenerative spinal disease
Coronado and George, 2018 [46]	USA	PSQ	To assess construct validity and concurrent validity of CSI and PSQ among patients with shoulder pain.	78 patients	Patients group = 39 ± 14.5, Control group = 35 ± 11.1	36 (46.2)	Chronic musculoskeletal pain	Shoulder pain
Carrillo-de-la-Peña et al., 2015 [74]	Spain	FSQ	To evaluate the extent of agreement between the 1990 and 2010 diagnostic criteria of the American College of Rheumatology (ACR) and to validate the Spanish adaptation of the FSQ.	65 patients	50 (22–65)	64 (98.5)	Mixed chronic pain	Fibromyalgia (FM)
Häuser et al., 2012 [75]	German	FSQ	To validate the German version of FSQ in fibromyalgia patients.	1651 patients	54 ± 9.8 (19–84)	1562 (94.8)	Mixed chronic pain	Fibromyalgia syndrome (FMS), chronic widespread pain
Fors et al., 2020 [76]	Norway	FSQ	To validate the Norwegian version of FSQ against the ACR1990 criteria.	120 patients and 62 controls	FM = 53 ± 10.9 Control = 48 ± 13	FM = 119 (99.2) Control = 59 (95.2)	Mixed chronic pain	Fibromyalgia
Jiao et al., 2023 [77]	China	FSQ	To assess the reliability and validity of the ACR 2011 and 2016 survey diagnostic criteria for fibromyalgia in China.	2 patients for pretesting, 200 FM patients and 200 RA patients (as control) for validation studies	FM group = 49 ± 13.4, RA group (Control) = 49 ± 13.2	FM = 174 (87.0) Control = 174 (87.0)	Mixed chronic pain	Fibromyalgia (FM *n* = 200), rheumatoid arthritis (RA *n* = 200)
Kang et al., 2019 [78]	Korea	FSQ	To validate the Korean version of the 2016 revised version of the 2010/2011 FSQ.	30 patients for pretesting, 86 FM and 89 other rheumatological disorders patients for validation studies	51 ± 11.8	135 (71.1)	Mixed chronic pain	Fibromyalgia (FM *n* = 86), rheumatoid arthritis (RA), systemic lupus erythematosus (SLE), osteoarthritis (OA), and myofascial pain syndrome (MPS) [all other disorders *n* = 89]
Moore et al., 2022 [79]	USA	FSQ	To examine the correlation between FSQ and QST as measures of pain centralization among patients with rheumatoid arthritis.	285 patients	55 ± 13.7	234 (82.1)	Chronic musculoskeletal pain	Rheumatoid arthritis (RA)
Aguirre Cárdenas et al., 2021 [80]	Chile	FSQ	To examine the psychometric properties of the Chilean version of the FSQ.	290 Patients and 117 participants without chronic pain	49 ± 14.3	407(100)	Mixed chronic pain	Fibromyalgia (FM *n* = 194), rheumatoid arthritis (RA *n* = 96)
Neville et al., 2018 [81]	USA	FSQ	To assess the convergent validity of FSQ in knee osteoarthritis patients.	129 patients	Female: 64 ± 8.6 Male: 65 ± 8.7	68 (52.7)	Chronic musculoskeletal pain	Knee osteoarthritis
Bidari et al., 2015 [82]	Iran	FSQ	To evaluate the validity of the Persian version of the FSQ and Polysymptomatic Distress Scale (PSD) in chronic pain patients.	263 patients	FM group = 42 ± 11 non-FM group = 48 ± 11	263(100)	Mixed chronic pain	Fibromyalgia (FM *n* = 169), osteoarthritis, periarthritis, regional pain syndromes [non-FM *n* = 94]
Ghavidel-Parsa et al., 2022 [83]	Iran	NFF	To develop and validate the preliminary NFF in patients with chronic pain.	185 patients	FM = 45 ± 9.9 NON-FM = 48 ± 11.5 (18–65)	185 (100)	Mixed chronic pain	Fibromyalgia, non-FM non-inflammatory chronic pain (osteoarthritis, tendonitis or painful periarticular conditions, mechanical low back pain, mechanical neck pain)
van Bemmel et al., 2019 [84]	Dutch	GPQ	To develop and evaluate the psychometric performance of this GPQ in patients with Fibromyalgia and rheumatoid arthritis.	212 patients	FM = 45 ± 11.6 RA = 60 ± 12.1	164 (77.4)	Mixed chronic pain	Fibromyalgia (FM *n* = 98), rheumatoid arthritis (RA *n* = 114)
Dixon et al., 2016 [85]	USA	SHS	To develop and validate the SHS.	Study 4: 124 patients and 66 healthy controls. Study 5: 5 patients and 44 healthy controls	Study 4: FM = 44 ± 11.3; Osteoarthritis = 59 ± 8.0, Osteoarthritis with FM = 56 ± 8.2, Healthy control = 35 ± 13.2 Study 5: Patient group = 41 ± 11.3 Control group = 40 ± 11.6	Study 4: 190 (100) Study 5: 51 (49.5)	Mixed chronic pain	Fibromyalgia, rheumatoid arthritis, chronic low back pain
Austin et al., 2020 [86]	Australia	novel self-report instrument	To develop and assess psychometric properties of a novel self-report questionnaire to assess symptoms associated with altered central nervous system pain processing in people with and without chronic pain.	99 patients and 84 healthy controls	Case = 57 ± 18.3 (19–89), Control = 41 ± 15.2	153 (83.6)	Mixed chronic pain	Osteoarthritis, Widespread pain, Neck and back pain, headaches/migraine, Abdominal/pelvic pain, Peripheral neuropathies, Radiculopathy
Ten Brink et al., 2021 [87]	Not reported	L-VISS and VDS	To validate L-VISS and VDS scale in chronic pain patients.	185 patients and 125 pain-free controls		251 (81)	Mixed chronic pain	Complex Regional Pain Syndrome (CRPS), fibromyalgia, general chronic pain

The mean age was rounded to the nearest whole number, rather than being displayed in decimal format. SD = Standard deviation, CSI = Central sensitization Inventory, PSQ = Pain Sensitivity Questionnaire, FSQ = Fibromyalgia Survey Questionnaire, NFF = Nociplastic-based Fibromyalgia Feature, GPQ = Generalized Pain Questionnaire, SHS = Sensory Hypersensitivity Scale, L-VISS and VDS = Leiden Visual Sensitivity Scale and Visual Discomfort Scale, PROM = Patient-reported outcome measure

**Table 5:** Summary of PROM identified

PROM	Developer	Subscales / Outcome domains	Number of items	Response options	Description	Range of scores/scoring	Original language	Available translations	Number of studies evaluating the instrument
Central Sensitization Inventory (CSI)	Mayer et al., 2012 [30]	4 factors: Physical functioning, Emotional Distress, Headache/Jaw Symptoms, Urological Symptoms	Two part- Part A: 25 items	(0–4)	Each of these items is measured on a 5-point temporal Likert scale, with the following numeric rating scale: never = 0, rarely = 1, sometimes = 2, often = 3, and always = 4.	Score range: 0 -100.	English	22	37
			Part B: 10 items (this part is for information only and is not scored)						
Pain Sensitivity Questionnaire (PSQ)	Ruscheweyh et al., 2009 [88]	Domain: Pain Sensitivity	17-items.	0–10	11-point scale with 0 meaning “not painful at all” 1 meaning ‘only just noticeable pain’ and 10 meaning “worst pain imaginable.” PSQ-total score = summed score of PSQ moderate (items: 1, 2, 4, 8, 15, 16, and 17) and PSQ minor (items: 3, 6, 7, 10, 11, 12, and 14) subscale or factor.	0–10.	German	6	8
Fibromyalgia Survey Questionnaire (FSQ)	Wolfe et al., 2010 [89]	2 subscales: Widespread pain distribution in designated body locations, severity of cognitive and presence of somatic symptoms	Symptom Severity Score (SS): 6 items -3 items on Severity of symptoms -3 items on somatic symptoms	0–3	3 items on Severity of symptoms each of which is scored by a Likert format from 0 (no problem) to 3 (severe: continuous, life-disturbing problems). 3 questions with a positive or negative response to somatic symptoms with a maximum score of 3.	Score range: 0–12.	English	7	9
			Widespread Pain Index (WPI): 19 number of painful body regions	0–1	Identifying 19 body areas with pain or tenderness.	Score range: 0–19.			
						A cumulative score range is 0–31 {(sum of the (0–19) WPI and the 6-item (0–12) SS scale)}. Fibromyalgia diagnostic criteria: (WPI ≥ 7 AND SS ≥ 5) OR (WPI 4– 6 AND SS ≥ 9).			
Nociplastic-based Fibromyalgia Features (NFF)	Ghavidel-Parsa et al., 2022 [83]	Domain: pain extent, migratory pain, pain aggravation with emotional or physical stress, affective component of pain perception, morning fatigue and pain hypersensitivity.	7 items	0–1	binary items with 1 = Yes and 0 = No responses	Cut-off score is 4	Persian		1
Generalized Pain Questionnaire (GPQ)	van Bemmel et al., 2019 [84]	Domain: pain sensitivity, after sensation, spreading of pain.	7- items	0–4	Each of these items is measured on a 5-point Likert-type rating scale with the following numeric rating scale: (0 = never, 1 = hardly noticed, 2 = moderately, 3 = strongly, 4 = very strongly	A cutoff score > 10 is suggested for identifying possible generalized pain hypersensitivity	Dutch		1
Sensory Hypersensitivity Scale (SHS)	Dixon et al., 2016 [85]	subscales: touch, taste, smell, hearing, light, pain, allergies, heat and cold.	25-item	0–5	5-point Likert-type rating scale: 1 = Strongly Disagree, 2 = Disagree, 3 = Neutral / Not Sure, 4 = Agree, 5 = Strongly agree		English		1
Novel self-report Questionnaire	Austin et al., 2020 [86]	2 clusters: pain symptoms, emotional and fatigue symptoms.	18-item	0–3	4-point Likert-type rating scale 0 = never, 1 = rarely, 2 = sometimes, 3 = always	Score range: 0–54 (factor 1 = 0–33, factor 2 = 0–21)	English		1
Leiden Visual Sensitivity Scale and Visual Discomfort Scale	Perenboom et al., 2018 [90]	Domains: Visual sensitivity to light and patterns	Leiden Visual Sensitivity Scale: 9-items scale	0–4	Each of these items is measured on a 5-point Likert scale to measure the degree of visual sensitivity, with the following numeric rating scale: not at all = 0, slightly = 1, moderately = 2, severely = 3, and very severe = 4.	Score range: 0–36	Dutch		1
	Conlon et al., 1999 [91]	Domains: somatic (e.g., being bothered, sore eyes), perceptual (e.g., afterimages, flickering, shimmering), and performance difficulties (e.g., worse eyesight, blurring) to different light sources or patterns.	Visual Discomfort Scale (VDS): 23 items	0–3	Each of these items is measured on a scale from 0 = (Event never occurs), 1= (Occasionally. A couple of times a year), 2= (Often. Every few weeks), to 3= (Almost always)	Score range: 0–69	English		

PROM = Patient-reported outcome measure, Ref = Reference

**Table 7:** Summary of COSMIN overall ratings of each measurement property of each PROM

PROMs	Content validity		Structural validity	Internal consistency	Cross-cultural validity	Reliability	Measurement error	Criterion validity	Hypothesis testing for construct validity	Responsiveness
	+/−/±/?**		+/−/±/?**	+/−/±/?**	+/−/±/?**	+/−/±/?**	+/−/±/?**	+/−/±/?**	+/−/±/?**	+/−/±/?**
CSI [30–66]	Relevance: (+) Comprehensiveness: (+) Comprehensibility: (+)	Overall (+)	Acceptable factor loading (+)	Cronbach’s α = 0.87–0.99 (+)	N/A	Test-retest ICC = 0.85–0.99 (+)	SEM = 0.31– 4.14; SDC (MDC) = 0.86 − 11.5 (MIC not determined) (?)	N/A	> 75% of the results aligned with the hypotheses (+)	Results in line with two hypotheses (+)
PSQ [46, 67–73]	Relevance: (+) Comprehensiveness: (+) Comprehensibility: (+)	Overall (+)	Acceptable factor loading (+)	Cronbach’s α = 0.87–0.96 (+)	N/A	Test-retest ICC = 0.71–0.93 (+)	N/A	N/A	> 25% of the results were not aligned with the hypotheses (-)	N/A
FSQ [74–82]	Relevance: (+) Comprehensiveness: (+) Comprehensibility: (+)	Overall (+)	N/A	Cronbach’s α = 0.71–0.94 (?)	N/A	Test-retest ICC = 0.79–0.86 (+)	N/A	N/A	> 75% of the results aligned with the hypotheses (+)	N/A
NFF [83]	N/A		N/A	Cronbach’s α = 0.72 (?)	N/A		N/A	AUC 0.87, sensitivity 82.5% and specificity 91.5% (+)	100% of the results aligned with the hypothesis (+)	N/A
GPQ [84]	N/A		Mokken analysis: Adequate model fit. (+)	Reliability coefficient *r* = 0.90 (+)	N/A	N/A	N/A	N/A	100% of the results aligned with the hypothesis (+)	N/A
SHS [85]	N/A		N/A	Cronbach’s alpha: SHS (total) = 0.86, SHS(factors) = 0.62–0.88 (?)	N/A	N/A	N/A		> 25% of the results were not aligned with the hypotheses (-)	N/A
Novel Self-report Instrument [86]	N/A		Acceptable factor loading (+)	Cronbach’s α: factor 1 = 0.94, factor 2 = 0.90 (+)	N/A	N/A	N/A	N/A	100% of the results aligned with the hypothesis (+)	N/A
L-VISS and VDS [87]	N/A		N/A	Cronbach’s α: L-VISS = 0.85, VDS = 0.94 (?)	N/A	N/A	N/A	N/A	100% of the results aligned with the hypothesis (+)	N/A

** + = sufficient, - = insufficient, ± = inconsistent,?= indeterminate; PROM = Patient-reported outcome measure, CSI = Central sensitization Inventory, PSQ = Pain Sensitivity Questionnaire, FSQ/FSDC = Fibromyalgia Survey Questionnaire/ Fibromyalgia Survey diagnostic criteria, NFF = Nociplastic-based Fibromyalgia Feature, GPQ = Generalized pain questionnaire, SHS = Sensory Hypersensitivity Scale, L-VISS and VDS = Leiden Visual Sensitivity Scale and Visual Discomfort Scale, N/A = not applicable

**Table 8:** Grading of the quality of evidence for measurement properties of proms

Measurement properties											
PROM	Content validity			Structural validity	Internal consistency	Cross-cultural validity	Reliability	Measurement error	Criterion validity	Hypothesis testing	Responsiveness
	Relevance	Comprehensiveness	Comprehensibility								
CSI [30–66]	**Moderate**	**Moderate**	**Moderate**	**Moderate**	**High**	N/A	**High**	N/A	N/A	**High**	**High**
PSQ [46,67–73]	**Moderate**	**Moderate**	**Moderate**	**Low**	**High**	N/A	**Low**	N/A	N/A	**High**	N/A
FSQ [74–82]	**Moderate**	**Moderate**	**Moderate**	N/A	**High**	N/A	**Moderate**	N/A	N/A	**High**	N/A
NFF [83]	N/A	N/A	N/A	N/A	**High**	N/A	N/A	N/A	**High**	**High**	N/A
GPQ [84]	N/A	N/A	N/A	**Very low**	**High**	N/A	N/A	N/A	N/A	**Moderate**	N/A
SHS [85]	N/A	N/A	N/A	N/A	**High**	N/A	N/A	N/A	N/A	**Moderate**	N/A
Novel Self-Report Instrument [86]	N/A	N/A	N/A	**Low**	**High**	N/A	N/A	N/A	N/A	**Low**	N/A
L-VISS and VDS [87]	N/A	N/A	N/A	N/A	**High**	N/A	N/A	N/A	N/A	**Low**	N/A

PROM = Patient-reported outcome measure, CSI = Central sensitization Inventory, PSQ = Pain Sensitivity Questionnaire, FSQ/FSDC = Fibromyalgia Survey Questionnaire/ Fibromyalgia Survey diagnostic criteria, NFF = Nociplastic-based Fibromyalgia Feature, GPQ = Generalized pain questionnaire, SHS = Sensory Hypersensitivity Scale, L-VISS and VDS = Leiden Visual Sensitivity Scale and Visual Discomfort Scale, NA = not applicable

### Meta-analysis

**Fig. 3:**
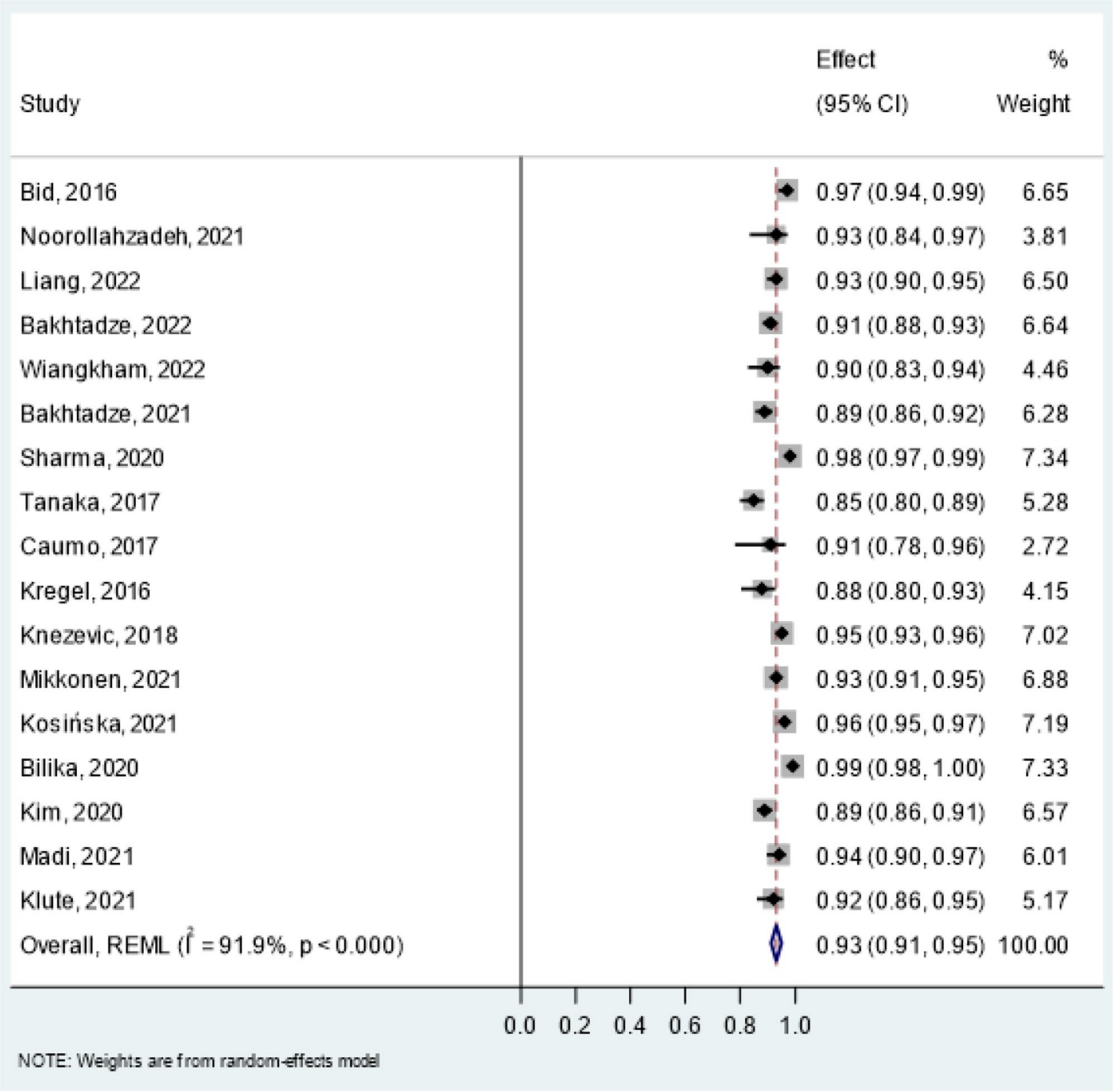
Forest Plot of pooled Intraclass Correlation Coefficients (ICC) of CSI in overall chronic pain conditions

**Fig. 4:**
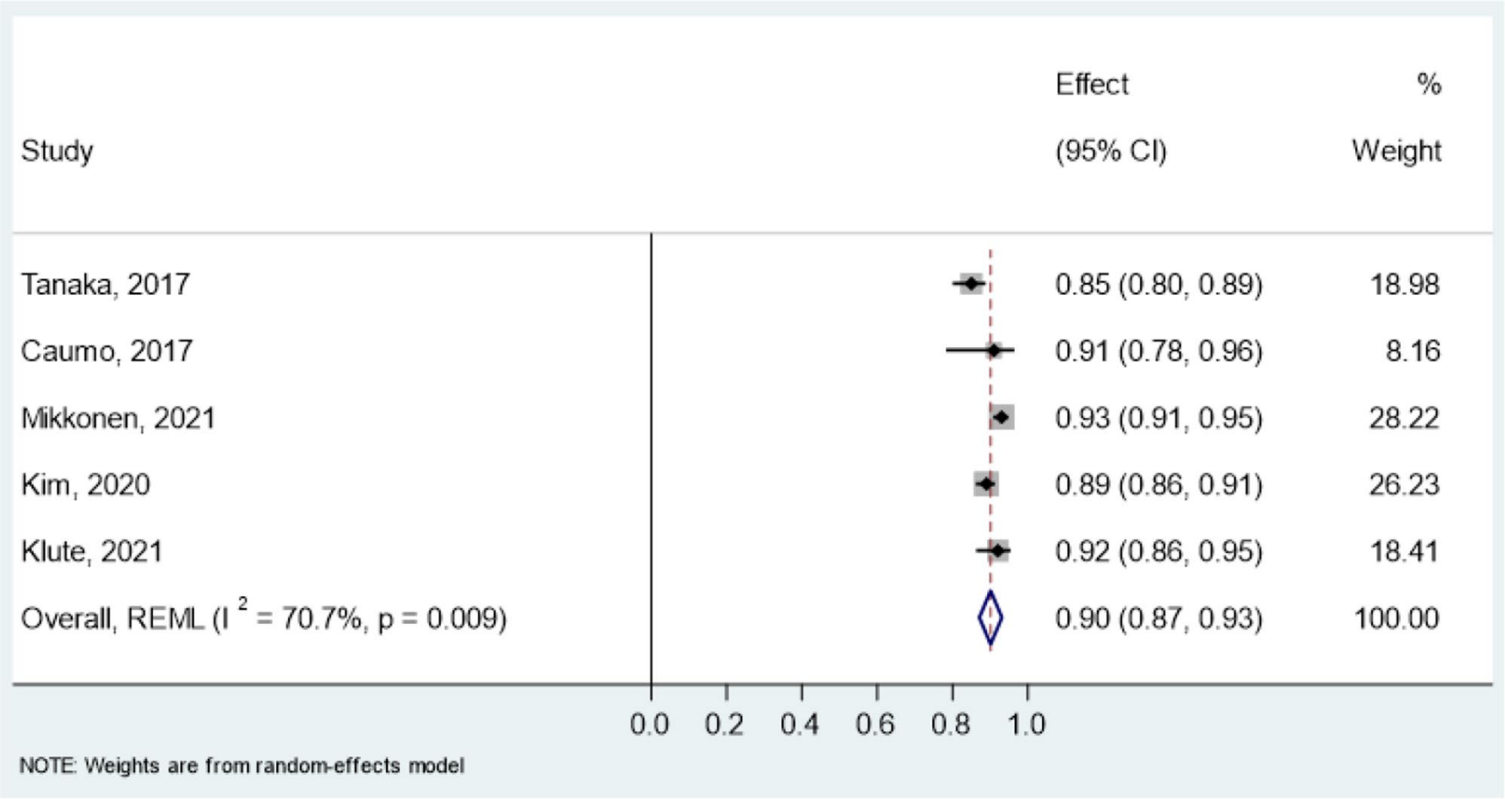
Forest Plot of pooled Intraclass Correlation Coefficients (ICC) of CSI in chronic musculoskeletal pain

**Fig. 5:**
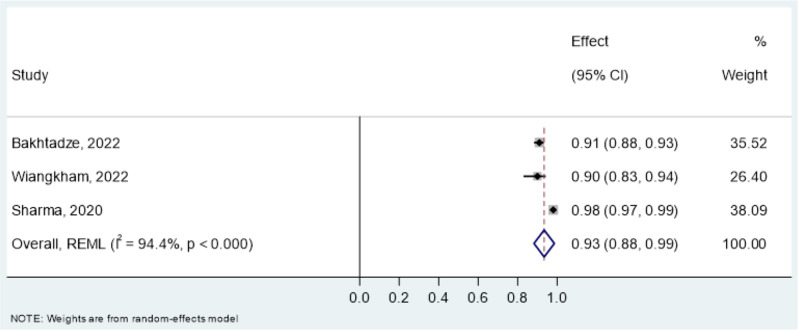
Forest Plot of pooled Intraclass Correlation Coefficients (ICC) of CSI in chronic neck pain

**Fig. 6:**
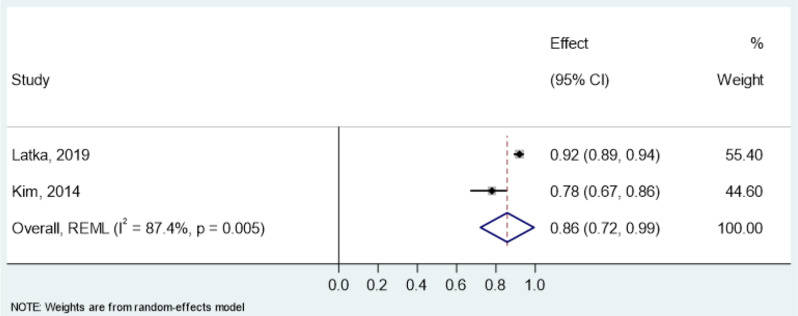
Forest Plot of pooled Intraclass Correlation Coefficients (ICC) of PSQ in chronic pain

Reliability estimates- intraclass correlation coefficient (ICC) for the CSI and PSQ were pooled in a meta-analysis (Table 9). All studies assessed test-retest reliability using mostly two-way random effects models with absolute agreement and single-rater type. For the CSI, pooled ICC was 0.93 (95% CI: 0.91–0.95, I² = 91.9%) in overall chronic pain conditions (Fig. 3), 0.90 (95% CI: 0.87–0.93, I² = 70.7%) in chronic musculoskeletal pain (Fig. 4) and 0.93 (95% CI: 0.88–0.99, I² = 94.4%) in chronic neck pain (Fig. 5). The PSQ showed an ICC of 0.86 (95% CI: 0.72–0.99, I² = 87.4%) in chronic pain conditions (Fig. 6). Due to insufficient data, meta-analysis of reliability estimates for other instruments was not conducted. Furthermore, publication bias was not assessed because there are no accepted methods for doing so yet in studies of systematic reviews of measurement properties of PROMs.

**Table 9:** Pooled results of reliability estimates in a meta-analysis

PROM	Conditions	Studies	ICC	95% CI	Cochrane’s Q	*P*-value	I^2^	τ²
CSI	Overall chronic pain (17 studies: *n* = 1825)	[31–33,35,36,38,39,42,44, 45,49,50,52,53,55,59,62]	0.93	0.91–0.95	196.92	< 0.0001	91.90%	0.0012
	Chronic musculoskeletal pain (5 studies: *n* = 684)	[42,44,50,55,62]	0.9	0.87–0.93	13.64	0.009	70.70%	0.0007
	Chronic non-specific neck pain (3 studies: *n* = 354)	[35,36,39]	0.93	0.88–0.99	35.67	< 0.0001	94.40%	0.0018
PSQ	Chronic pain (2 studies: *n* = 216)	[71,73]	0.86	0.72–0.99	7.96	0.005	87.40%	0.0086

PROM = Patient reported outcome measures, CSI = Central sensitization Inventory, PSQ = Pain Sensitivity Questionnaire, ICC = Intraclass correlation coefficient

## Discussion

This systematic review identified eight PROMs used to assess CS manifestations in patients with chronic pain conditions. Some measurement instruments were not originally developed to assess CS pain, like L-VISS & VDS [90, 91]. However, these instruments were tested in chronic pain patients and could be used to assess CS-related symptoms (e.g., sensory hyperresponsiveness to light and patterns) [87]. Not all the instruments were designed to assess all the core features (pain intensity, sensory hyperresponsiveness, physical well-being, pain interference with functionality, and psychological distress) associated with CS. For example, PSQ [88] focuses only on pain intensity and pain threshold to external stimuli. However, it is recommended to incorporate all PROMs that assess one or more specific constructs of interest, instead of solely prioritizing the most frequently utilized PROMs [22, 25]. The findings of this review showed that most identified PROMs have limited evidence regarding their psychometric properties. Again, the lack of sufficient evidence for most instruments does not necessarily indicate poor instrument quality. Instead, it predominantly reflects the limited availability of published articles as well as the poor methodological quality of studies examining these properties.

For many of the included studies, the rating of methodological quality of studies was doubtful or inadequate. This is due to inadequate sample size or due to unclear reporting of important information, which downgraded their evaluations. For example, almost all the studies assessing content validity [31–34, 36, 39, 44, 49, 50, 57, 59, 63, 69, 71, 73, 74, 80] got a doubtful rating due to a lack of adequate information (e.g., sample size, use of appropriate methods to engage patients, qualifications of interviewers or moderators, use of interview guides, transcription procedures, data analysis approaches and so on). In a few included studies, factor analyses were absent, despite the recommendation to assess the unidimensionality of the instrument before computing internal consistency values (i.e. Cronbach’s alpha) [31, 39, 53, 74–78, 80, 82]. Given that the target population in this review consisted of individuals with chronic pain conditions, a time interval of 5 days to 2 weeks was considered appropriate for test-retest reliability assessments for addressing both the requirement to minimize recall bias and to ensure the stability of patients’ conditions. The time gap between the test and retest did not align with the predetermined criteria for assessing reliability in certain studies [35, 36, 38, 45, 47, 67, 71, 73].

A total of forty-two studies [31–33, 35–42, 44, 45, 47–50, 52–57, 59–63, 66, 68–78, 80, 82] evaluated the translated version of the PROMs, but cross-cultural validity has not yet been assessed. Assessment of cross-cultural validity is crucial in translation studies to determine if the translated versions evaluate culturally different populations similarly to their original counterparts. The evaluation of measurement error is fundamental to differentiate actual changes from both systematic and random errors. In this review, twelve studies were found to assess measurement error [31, 35, 36, 38, 39, 48–50, 52, 53, 59, 62], although the MIC value was not determined, which signifies a meaningful change in a patient’s condition. Without specifying the MIC, evidence of measurement error remains indeterminate, regardless of the proper calculation of the smallest detectable change. Also, inadequate and doubtful ratings of the quality of some studies for hypothesis testing for construct validity resulted from the absence of a clear description of the comparator instruments [35, 38, 47, 71, 86, 87]. For responsiveness, only two studies were identified [65, 66] to detect the change in the assessment score following the interventions received by patients. A few studies were found to report missing data [30, 33, 44, 45, 47, 55]. When there was a greater percentage of missing data (more than 20% of total items), we downgraded the rating of the quality of measurement property (structural validity) of instruments. While there is no universally agreed threshold, we based our decision on guidance from methodologists who suggest that > 20% missing data may pose a risk of bias [92].

Among the eight instruments assessed, CSI was assessed most frequently and it has the most evidence to support psychometric properties in the target indication. In particular, seven out of nine measurement properties of CSI were evaluated [30–66]. The overall ratings of six measurement properties were sufficient with moderate to high evidence. Confirmatory factor analysis and/or exploratory factor analysis confirmed the adequacy of structural validity of CSI with acceptable factor loadings. The findings from hypothesis testing for construct validity indicated that CSI demonstrates a strong correlation with other PROMs assessing related but dissimilar constructs. Moreover, CSI was found to be responsive to changes following interventions. Based on 17 studies, the pooled results of the test-retest reliability of the CSI demonstrated excellent reliability for overall chronic pain conditions, with an ICC of 0.93 (95% CI: 0.91–0.95). Despite this high reliability, there was substantial heterogeneity, as indicated by an I² statistic of 91.9%. The variability in the observed heterogeneity could be attributed to several factors, including differences in the study populations, such as the types and severity of chronic pain conditions, as well as variations in the study’s time and location. The PSQ was found to assess four measurement properties, having sufficient overall ratings with low to high levels of evidence [46, 67–73]. Based on the pooled result, PSQ was found to have excellent reliability with an ICC of 0.86 (95% CI: 0.72–0.99) and high heterogeneity I^2^ = 87.4% in chronic pain conditions. The FSQ was found to have sufficient overall ratings for three properties and an indeterminate rating for one measurement property (internal consistency) despite having high-level evidence [74–82]. The rest of the PROMs had very low to high evidence with sufficient ratings for two to three measurement properties.

Selection of an appropriate PROM also involves considering other aspects like feasibility and interpretability. These are considered non-quantifiable attributes since they do not directly assess the quality of a PROM [25]. This review encountered difficulties in comparing the interpretability of the identified PROMs because the included studies lacked sufficient information. However, few studies have reported the absence of floor and ceiling effects for CSI [33–35, 37, 50, 53, 57, 62], PSQ [71, 73] and L-VSS &VDS [87]. Floor or ceiling effects are considered present when more than 15% of respondents achieve the lowest or highest possible score, respectively [93].

### Strengths and limitations

The present systematic review has several strengths. First, we identified all available PROMs used to assess central sensitization manifestations in patients with chronic pain. Next, a thorough and systematic evaluation of both the methodological quality of studies and the quality of psychometric properties of PROMs was conducted, with the results of studies being pooled quantitatively where possible. Thus, this review provides a broad and detailed overview of the quality of available PROMs. While Scerbo and colleagues [94] assessed only the central sensitization inventory (CSI) in a systematic review, the present review encompasses more instruments and uses the most updated recommendations (COSMIN) of quality assessment.

This review has some limitations. The rating system used to assess instruments may be prone to subjective biases, which could potentially affect the consistency of evaluations. To mitigate this, two reviewers independently assessed the studies, with conflicts resolved by experts, ensuring decisions were consensus-based. Another limitation is the risk of language bias, as only English-language studies were included, excluding three studies on CSI and PSQ published in other languages, which could have influenced the findings. The findings of this review are based solely on studies involving adult populations and may not be generalizable to pediatric populations.

### Recommendations for future research

This review highlights a significant research gap in assessing measurement properties of included PROMs, as none of the PROMs have been assessed in all nine measurement properties. Future studies are needed to prioritize the comprehensive evaluation of the fundamental psychometric properties of all these instruments, ensuring adherence to recommended validation guidelines.

In this review, there is evidence of poor reporting of many of the included studies. Therefore, we suggest that the studies assessing the measurement properties of PROMs place greater emphasis on following standardized reporting guidelines such as COSMIN reporting guidelines [95] to ensure transparency and accessibility of relevant information.

Numerous studies have identified core features of central sensitization [9, 10, 96–98]. Future research should develop standardized core outcome domains designed to assess chronic pain with central sensitization which will promote greater consistency and comparability among studies within this area.

Furthermore, our findings suggest that if future guidelines were to offer additional clarity on best practices for reporting parameter estimates of measurement properties and on outlining meta-analytic procedures, this could potentially help enhance the robustness and comparability of quantitative syntheses in this field.

## Conclusion

This systematic review provided a summary of the psychometric quality of eight identified instruments based on available publications. The findings of this review could provide valuable guidance to healthcare providers and researchers to select appropriate tools for assessing treatment outcomes and making clinical decisions accordingly for providing patient-centred care in chronic pain patients with central sensitization. The CSI was found to receive the highest overall ratings with moderate to high levels of quality of evidence among all the included instruments. The other PROMs included did not achieve similarly high and sufficient ratings due to numerous incomplete evaluations as well as a lack of publications. Although not all properties have been studied, the CSI could serve as a useful PROM for chronic pain associated with central sensitization. Careful consideration is advised when selecting an instrument, as none of the included PROMs had sufficient evidence across all nine measurement properties.

## Electronic supplementary material

Below is the link to the electronic supplementary material.

Supplementary Material 1

Supplementary Material 2

Supplementary Material 3

## Data Availability

All data supporting the findings of this study are available within the paper and its Supplementary Information. The protocol for this review can be found on PROSPERO (CRD42023460050).
