# Supplementary material for: Psychometric properties of patient-reported outcome measures in chronic pain conditions with central sensitization- a systematic review and meta-analysis
Source: J Patient Rep Outcomes. 2025 Jul 11;9:87. doi: 10.1186/s41687-025-00919-9 (PMC12254461; doi:10.1186/s41687-025-00919-9)
Supplement: Supplementary file 1 — Supplementary Material 1 [file 41687_2025_919_MOESM1_ESM.docx]

**Appendix A: Search Strategy**

**Databases: Search performed on September 30, 2023**

**PubMed *192 records***

**Measurement properties filter:**

(instrumentation[sh] OR methods[sh] OR “Validation Studies”[pt] OR “Comparative Study”[pt] OR “psychometrics”[MeSH] OR psychometr*[tiab] OR clinimetr*[tw] OR clinometr*[tw] OR “outcome assessment (health care)”[MeSH] OR “outcome assessment”[tiab] OR “outcome measure*”[tw] OR “observer variation”[MeSH] OR “observer variation”[tiab] OR “Health Status Indicators”[Mesh] OR “reproducibility of results”[MeSH] OR reproducib*[tiab] OR “discriminant analysis”[MeSH] OR reliab*[tiab] OR unreliab*[tiab] OR valid*[tiab] OR “coefficient of variation”[tiab] OR coefficient[tiab] OR homogeneity[tiab] OR homogeneous[tiab] OR “internal consistency”[tiab] OR (cronbach*[tiab] AND (alpha[tiab] OR alphas[tiab])) OR (item[tiab] AND (correlation*[tiab] OR selection*[tiab] OR reduction*[tiab])) OR agreement[tw] OR precision[tw] OR imprecision[tw] OR “precise values”[tw] OR test-retest[tiab] OR (test[tiab] AND retest[tiab]) OR (reliab*[tiab] AND (test[tiab] OR retest[tiab])) OR stability[tiab] OR interrater[tiab] OR inter-rater[tiab] OR intrarater[tiab] OR intra-rater[tiab] OR intertester[tiab] OR inter-tester[tiab] OR intratester[tiab] OR intra-tester[tiab] OR interobserver[tiab] OR inter-observer[tiab] OR intraobserver[tiab] OR intra-observer[tiab] OR intertechnician[tiab] OR inter-technician[tiab] OR intratechnician[tiab] OR intra-technician[tiab] OR interexaminer[tiab] OR inter-examiner[tiab] OR intraexaminer[tiab] OR intra-examiner[tiab] OR interassay[tiab] OR inter-assay[tiab] OR intraassay[tiab] OR intra-assay[tiab] OR interindividual[tiab] OR inter-individual[tiab] OR intraindividual[tiab] OR intra-individual[tiab] OR interparticipant[tiab] OR inter-participant[tiab] OR intraparticipant[tiab] OR intra-participant[tiab] OR kappa[tiab] OR kappa’s[tiab] OR kappas[tiab] OR repeatab*[tw] OR ((replicab*[tw] OR repeated[tw]) AND (measure[tw] OR measures[tw] OR findings[tw] OR result[tw] OR results[tw] OR test[tw] OR tests[tw])) OR generaliza*[tiab] OR generalisa*[tiab] OR concordance[tiab] OR (intraclass[tiab] AND correlation*[tiab]) OR discriminative[tiab] OR “known group”[tiab] OR “factor analysis”[tiab] OR “factor analyses”[tiab] OR “factor structure”[tiab] OR “factor structures”[tiab] OR dimension*[tiab] OR subscale*[tiab] OR (multitrait[tiab] AND scaling[tiab] AND (analysis[tiab] OR analyses[tiab])) OR “item discriminant”[tiab] OR “interscale correlation*”[tiab] OR error[tiab] OR errors[tiab] OR “individual variability”[tiab] OR “interval variability”[tiab] OR “rate variability”[tiab] OR (variability[tiab] AND (analysis[tiab] OR values[tiab])) OR (uncertainty[tiab] AND (measurement[tiab] OR measuring[tiab])) OR “standard error of measurement”[tiab] OR sensitiv*[tiab] OR responsive*[tiab] OR (limit[tiab] AND detection[tiab]) OR “minimal detectable concentration”[tiab] OR interpretab*[tiab] OR ((minimal[tiab] OR minimally[tiab] OR clinical[tiab] OR clinically[tiab]) AND (important[tiab] OR significant[tiab] OR detectable[tiab]) AND (change[tiab] OR difference[tiab])) OR (small*[tiab] AND (real[tiab] OR detectable[tiab]) AND (change[tiab] OR difference[tiab])) OR “meaningful change”[tiab] OR “ceiling effect”[tiab] OR “floor effect”[tiab] OR “Item response model”[tiab] OR IRT[tiab] OR Rasch[tiab] OR “Differential item functioning”[tiab] OR DIF[tiab] OR “computer adaptive testing”[tiab] OR “item bank”[tiab] OR “cross-cultural equivalence”[tiab])

AND

("Central sensitization") OR ("central pain") OR ("centralized pain") OR ("nociplastic pain") OR ("pain sensitization") OR ("pain hypersensitivity")OR ("central hyperexcitability") OR ("heightened pain sensitivity")

AND

("chronic pain") OR ("chronic primary pain condition")) OR ("central pain syndrome")) OR ("central sensitivity syndrome")) OR ("central sensitization syndrome")) OR ("Functional somatic syndrome")) OR ("chronic pain condition")) OR ("chronic musculoskeletal pain")

AND

("patient reported") OR ("self reported")) OR ("self administered")) OR ("patient administered")) OR ("measure")

AND

("Questionnaire") OR ("tool")) OR ("scale")) OR ("instrument")) OR ("measurement")) OR ("Assessment")) OR ("index") OR ("central sensitization inventory") OR ("CSI")) OR ("Pain sensitivity questionnaire")) OR ("PSQ")) OR ("Fibromyalgia survey questionnaire"))

**Exclusion filter**

(“addresses”[Publication Type] OR “biography”[Publication Type] OR “case reports”[Publication Type] OR “comment”[Publication Type] OR “directory”[Publication Type] OR “editorial”[Publication Type] OR “festschrift”[Publication Type] OR “interview”[Publication Type] OR “lectures”[Publication Type] OR “legal cases”[Publication Type] OR “legislation”[Publication Type] OR “letter”[Publication Type] OR “news”[Publication Type] OR “newspaper article”[Publication Type] OR “patient education handout”[Publication Type] OR “popular works”[Publication Type] OR “congresses”[Publication Type] OR “consensus development conference”[Publication Type] OR “consensus development conference, nih”[Publication Type] OR “practice guideline”[Publication Type]) NOT (“animals”[MeSH Terms] NOT “humans”[MeSH Terms])

[**MEDLINE (Ovid)**](https://www.lib.uwo.ca/cgi-bin/ezpauthn.cgi?url=http://ovidsp.ovid.com/ovidweb.cgi?T=JS&PAGE=main&D=medall) ***554 records***

1.

(Central sensitization* or centralized pain* or central pain* or nociplastic pain* or pain sensitization* or pain hypersensitivity* or central hyperexcitability* or heightened pain sensitivity*).tw,kf.

2.

(chronic pain* or chronic primary pain* or central pain syndrome* or central sensitivity syndrome* or central sensitization syndrome* or Functional somatic syndrome* or chronic pain condition* or chronic musculoskeletal pain*).tw,kf.

3.

(patient reported or self reported or self administered or patient administered or measure).tw,kf.

4.

(Questionnaire or tool or scale or instrument or measurement or index).tw,kf.

5.

(central sensitization inventory or CSI).tw,kf.

6.

"pain sensitivity questionnaire*".tw,kf.

7.

"Fibromyalgia survey questionnaire*".tw,kf.

8.

(Widespread Pain Index and Symptom Severity Scale).tw,kf.

9.

(Leiden Visual Sensitivity Scale and Visual Discomfort Scale).tw,kf.

10.

"Pain questionnaires*".tw,kf.

11.

"Pain instruments*".tw,kf.

12.

Pain Measurement*.mp.

13.

Pain Assessment*.mp.

14.

Pain scale*.mp.

15.

(Pain adj3 tool*).tw,kf.

16.

(pain adj3 questionnaire*).tw,kf.

17.

(checklist adj3 pain*).tw,kf.

18.

(instrumentation or methods).mp.

19.

outcome measure*.mp.

20.

outcome assessment*.tw.

21.

exp Psychometrics/

22.

"psychometr*".tw,kf.

23.

"Measurement properties*".tw,kf.

24.

(clinimetr* or clinometr*).tw,kf.

25.

"Measurement error*".tw,kf.

26.

Internal consistency*.mp.

27.

Reproducibility of result*.mp.

28.

reproducib*.tw.

29.

Reliability*.mp.

30.

(reliab* or unreliab* or valid* or coefficient of variation or coefficient or homogeneity or homogeneous or internal consistency).tw,kf.

31.

(cronbach* and (alpha or alphas)).tw.

32.

validity*.mp.

33.

validation studies*.tw,kf.

34.

comparative study*.mp.

35.

(item and (correlation* or selection* or reduction*)).tw.

36.

(precision or imprecision or precise values).mp.

37.

(test-retest or (test and retest) or (reliab* and (test or retest))).tw,kf.

38.

(interrater or inter-rater or intrarater or intra-rater or intertester or inter-tester or intratester or intra-tester or interobserver or inter-observer or intraobserver or intra-observer).tw,kf.

39.

(intertechnician or inter-technician or intratechnician or intra-technician or interexaminer or inter-examiner or intraexaminer or intra-examiner or interassay or inter-assay or intraassay or intra-assay).tw,kf.

40.

(interindividual or inter-individual or intraindividual or intra-individual or interparticipant or inter-participant or intraparticipant or intra-participant).tw.

41.

(kappa or kappas).tw,kf.

42.

repeatab*.mp.

43.

((replicab* or repeated) and (measure or measures or findings or result or results or test or tests)).tw.

44.

hypothesis testing*.mp.

45.

Generalizability*.mp.

46.

(generaliza* or generalisa*).tw.

47.

(intraclass and correlation*).tw.

48.

(factor analysis or factor analyses or factor structure or factor structures).tw.

49.

(dimension or subscale).mp.

50.

(multitrait and scaling and (analysis or analyses)).tw.

51.

(item discriminant or interscale correlation).mp.

52.

interscale correlation*.tw.

53.

(error or errors).tw.

54.

(individual variability or interval variability).tw.

55.

rate variability.tw.

56.

(variability and (analysis or values)).tw.

57.

(uncertainty and (measurement or measuring)).tw.

58.

standard error of measurement.tw.

59.

(sensitiv or responsive).tw.

60.

(limit and detection).mp.

61.

minimal detectable concentration.tw.

62.

interpretab*.tw.

63.

((minimal or minimally or clinical or clinically) and (important or significant or detectable) and (change or difference)).tw.

64.

(small* and (real or detectable) and (change or difference)).tw.

65.

meaningful change.tw.

66.

(ceiling effect or floor effect).tw.

67.

item response model.tw.

68.

IRT.tw.

69.

rasch.tw.

70.

differential item functioning.tw.

71.

DIF.tw.

72.

computer adaptive testing.tw.

73.

item bank.tw.

74.

cross-cultural equivalence.tw.

75.

1 and 2

76.

3 or 4 or 5 or 6 or 7 or 8 or 9 or 10 or 11 or 12 or 13 or 14 or 15 or 16 or 17

77.

18 or 19 or 20 or 21 or 22 or 23 or 24 or 25 or 26 or 27 or 28 or 29 or 30 or 31 or 32 or 33 or 34 or 35 or 36 or 37 or 38 or 39 or 40 or 41 or 42 or 43 or 44 or 45 or 46 or 47 or 48 or 49 or 50 or 51 or 52 or 53 or 54 or 55 or 56 or 57 or 58 or 59 or 60 or 61 or 62 or 63 or 64 or 65 or 66 or 67 or 68 or 69 or 70 or 71 or 72 or 73 or 74

78.

75 and 76 and 77

**EMBASE *157 records***

('Validation study' or 'feasibility study' or 'intermethod comparison' or 'data collection method' or 'psychometry' or 'reproducibility' or reproducib*:ab,ti or 'audit':ab,ti or psychometr*:ab,ti or clinimetr*:ab,ti or clinometr*:ab,ti or 'observer variatio' or 'observer variation':ab,ti or 'discriminant analysis' or 'validity' or reliab*:ab,ti or valid*:ab,ti or 'coefficient':ab,ti or 'internal consistency':ab,ti or (cronbach*:ab,ti and ('alpha':ab,ti or 'alphas':ab,ti)) or 'item correlation':ab,ti or 'item correlations':ab,ti or 'item selection':ab,ti or 'item selections':ab,ti or 'item reduction':ab,ti or 'item reductions':ab,ti or 'agreement':ab,ti or 'precision':ab,ti or 'imprecision':ab,ti or 'precise values':ab,ti or 'test-retest':ab,ti or ('test':ab,ti and 'retest':ab,ti) or (reliab*:ab,ti and ('test':ab,ti or 'retest':ab,ti)) or 'stability':ab,ti or 'interrater':ab,ti or 'inter-rater':ab,ti or 'intrarater':ab,ti or 'intra-rater':ab,ti or 'intertester':ab,ti or 'inter-tester':ab,ti or 'intratester':ab,ti or 'interobeserver':ab,ti or 'inter-observer':ab,ti or 'intraobserver':ab,ti or 'intertechnician':ab,ti or 'inter-technician':ab,ti or 'intratechnician':ab,ti or 'interexaminer':ab,ti or 'inter-examiner':ab,ti or 'intraexaminer':ab,ti or 'interassay':ab,ti or 'inter-assay':ab,ti or 'intraassay':ab,ti or 'intra-assay':ab,ti or 'interindividual':ab,ti or 'inter-individual':ab,ti or 'intraindividual':ab,ti or 'intra-individual':ab,ti or 'interparticipant':ab,ti or 'interparticipant':ab,ti or 'intraparticipant':ab,ti or 'kappa':ab,ti or 'kappas':ab,ti or 'coefficient of variation':ab,ti or repeatab*:ab,ti or ((replicab*:ab,ti or 'repeated':ab,ti) and ('measure':ab,ti or 'measures':ab,ti or 'findings':ab,ti or 'result':ab,ti or 'results':ab,ti or 'test':ab,ti or 'tests':ab,ti)) or generaliza*:ab,ti or generalisa*:ab,ti or 'concordance':ab,ti or ('intraclass':ab,ti and correlation*:ab,ti) or 'discriminative':ab,ti or 'known group':ab,ti or 'factor analysis':ab,ti or 'factor analyses':ab,ti or 'factor structure':ab,ti or 'factor structures':ab,ti or 'dimensionality':ab,ti or subscale*:ab,ti or 'multitrait scaling analysis':ab,ti or 'multitrait scaling analyses':ab,ti or 'item discriminant':ab,ti or 'interscale correlation':ab,ti or 'interscale correlations':ab,ti or (('error':ab,ti or 'errors':ab,ti) and (measure*:ab,ti or correlat*:ab,ti or evaluat*:ab,ti or 'accuracy':ab,ti or 'accurate':ab,ti or 'precision':ab,ti or 'mean':ab,ti)) or 'individual variability':ab,ti or 'interval variability':ab,ti or 'rate variability':ab,ti or 'variability analysis':ab,ti or ('uncertainty':ab,ti and ('measurement':ab,ti or 'measuring':ab,ti)) or 'standard error of measurement':ab,ti or sensitiv*:ab,ti or responsive*:ab,ti or ('limit':ab,ti and 'detection':ab,ti) or 'minimal detectable concentration':ab,ti or interpretab*:ab,ti or (small*:ab,ti and ('real':ab,ti or 'detectable':ab,ti) and ('change':ab,ti or 'difference':ab,ti)) or 'meaningful change':ab,ti or 'minimal important change':ab,ti or 'minimal important difference':ab,ti or 'minimally important change':ab,ti or 'minimally important difference':ab,ti or 'minimal detectable change':ab,ti or 'minimal detectable difference':ab,ti or 'minimally detectable change':ab,ti or 'minimally detectable difference':ab,ti or 'minimal real change':ab,ti or 'minimal real difference':ab,ti or 'minimally real change':ab,ti or 'minimally real difference':ab,ti or 'ceiling effect':ab,ti or 'floor effect':ab,ti or 'item response model':ab,ti or 'irt':ab,ti or 'rasch':ab,ti or 'differential item functioning':ab,ti or 'dif':ab,ti or 'computer adaptive testing':ab,ti or 'item bank':ab,ti or 'cross-cultural equivalence':ab,ti

AND

("chronic pain condition*" or "central sensitivity syndrome*" or "central pain syndrome*" or "chronic primary pain condition*" or "chronic pain*" or "chronic overlapping pain condition*" or "Functional somatic syndrome*" or "chronic musculoskeletal pain*").mp.

AND

("Central sensitization*" or "Central sensitisation*" or "centralized pain*" or "central pain*" or "nociplastic pain*" or "pain sensitization*" or "pain sensitivity*" or "pain hypersensitization*" or "central hyperexcitability*").mp.

AND

("pain assessment*" or "pain measurement*" or "pain rating*" or "disability*" or "quality of life*" or "outcome assessment*" or "quality-of-life*" or "functional assessment*" or "self report*" or "self evaluation*" or "self assessment*" or "self-assessment*" or "self-report*" or "self-reported*" or "self reported*" or "patient reported outcome*").mp.

OR

("Questionnaire*" or "tool*" or "scale*" or "instrument*" or "measurement*" or "index*").mp.

OR

("central sensitization inventory*" or "CSI*").mp.

OR

"pain sensitivity questionnaire*".mp.

OR

"Fibromyalgia survey questionnaire*".mp.

OR

"Widespread Pain Index and Symptom Severity Scale*".mp.

**Scopus *1162 records***

TITLE-ABS-KEY(psychometr*) OR TITLE-ABS-KEY(clinimetr*) OR TITLE-ABS-KEY(clinometr*) OR TITLE-ABS-KEY('observer') OR TITLE-ABS-KEY('observer variation') OR TITLE-ABS-KEY(reliab*) OR TITLE-ABS-KEY(valid*) OR TITLE-ABS-KEY('imprecision') OR TITLE-ABS-KEY('precise values') OR TITLE-ABS-KEY('coefficient') OR TITLE-ABS-KEY(reproducib*) OR TITLE-ABS-KEY('internal consistency') OR TITLE-ABS-KEY((cronbach*) AND ('alpha' OR 'alphas')) OR TITLE-ABS-KEY ('item correlation') OR TITLE-ABS-KEY('item correlations') OR TITLE-ABS-KEY('item selection') OR TITLE-ABS-KEY('item selections') OR TITLE-ABS-KEY('item reduction') OR TITLE-ABS-KEY('item reductions') OR TITLE-ABS-KEY('agreement') OR TITLE-ABS-KEY('precision') OR TITLE-ABS-KEY('test-retest') OR TITLE-ABS-KEY('test' AND 'retest') OR TITLE-ABS-KEY(reliab*) AND ('test' OR 'retest') OR TITLE-ABS-KEY('stability') OR TITLE-ABS-KEY('interrater') OR TITLE-ABS-KEY('inter-rater') OR TITLE-ABS-KEY('intrarater') OR TITLE-ABS-KEY('intra-rater') OR TITLE-ABS-KEY('intertester') OR TITLE-ABS-KEY('inter-tester') OR TITLE-ABS-KEY('intratester') OR TITLE-ABS-KEY('intratester') OR TITLE-ABS- KEY('interobserver') OR TITLE-ABS-KEY('intertechnician') OR TITLE-ABS-KEY('inter-technician') OR TITLE-ABS-KEY('intratechnician') OR TITLE-ABS-KEY('intratechnician') OR TITLE-ABS-KEY('inter-observer') OR TITLE-ABS-KEY('intraobserver') OR TITLE-ABS-KEY('intraobserver') OR TITLE-ABS-KEY('interexaminer') OR TITLE-ABS-KEY('inter-examiner') OR TITLE-ABS-KEY('intraexaminer') OR TITLE-ABS-KEY('intraexaminer') OR TITLE-ABS-KEY('interassay') OR TITLE-ABS-KEY('inter-assay') OR TITLE-ABS-KEY('intraassay') OR TITLE-ABS-KEY('intra-assay') OR TITLE-ABS-KEY('interindividual') OR TITLE-ABS-KEY('interindividual') OR TITLE-ABS-KEY('intraindividual') OR TITLE-ABS-KEY('intra-individual') OR TITLE-ABS-KEY('interparticipant') OR TITLE-ABS-KEY('inter-participant') OR TITLE-ABS-KEY('intraparticipant') OR TITLE-ABS-KEY('intraparticipant') OR TITLE-ABS-KEY('kappa') OR TITLE-ABS-KEY('kappas') OR TITLE-ABS-KEY('coefficient of variation') OR TITLE-ABS-KEY(repeatab*) OR TITLE-ABS-KEY((replicab* OR 'repeated') AND ('measure' OR 'measures' OR 'findings' OR 'result' OR 'results' OR 'test' OR 'tests')) OR TITLE-ABS-KEY(generaliza* OR generalisa*) OR TITLE-ABS-KEY('concordance') OR TITLE-ABS-KEY('intraclass' AND correlation) OR TITLE-ABS-KEY('discriminative') OR TITLE-ABS-KEY('known group') OR TITLE-ABS-KEY('factor analysis') OR TITLE-ABS-KEY('factor analyses') OR TITLE-ABS-KEY('factor structure') OR TITLE-ABS-KEY('factor structures') OR TITLE-ABS-KEY('dimensionality') OR TITLE-ABS-KEY(subscale*) OR TITLE-ABS-KEY('multitrait scaling analysis') OR TITLE-ABS-KEY('multitrait scaling analyses') OR TITLE-ABS-KEY('item discriminant') OR TITLE-ABS-KEY('interscale correlation') OR TITLE-ABS-KEY('interscale correlations') OR TITLE-ABS-KEY(('error' OR 'errors') AND (measure OR correlat* OR evaluat* OR 'accuracy' OR 'accurate' OR 'precision' OR 'mean')) OR TITLE-ABS-KEY('individual variability') OR TITLE-ABS-KEY('interval variability') OR TITLE-ABS-KEY('rate variability') OR TITLE-ABS-KEY('variability analysis') OR TITLE-ABS-KEY('uncertainty' AND ('measurement' OR 'measuring')) OR TITLE-ABS-KEY('standard error of measurement') OR TITLE-ABS-KEY(sensitiv*) OR TITLE-ABS-KEY(responsive*) OR TITLE-ABS-KEY('limit' AND 'detection') OR TITLE-ABS-KEY('minimal detectable concentration') OR TITLE-ABS-KEY(interpretab*) OR TITLE-ABS-KEY(small* AND ('real' OR 'detectable') AND ('change' OR 'difference')) OR TITLE-ABS-KEY('meaningful change') OR TITLE-ABS-KEY('minimal important change') OR TITLE-ABS-KEY('minimal important difference') OR TITLE-ABS-KEY('minimally important change') OR TITLE-ABSKEY('minimally important difference') OR TITLE-ABS-KEY('minimal detectable change') OR TITLE-ABS-KEY('minimal detectable difference') OR TITLE-ABS-KEY('minimally detectable change') OR TITLE-ABS-KEY('minimally detectable difference') OR TITLE-ABS-KEY('minimal real change') OR TITLE-ABS-KEY('minimal real difference') OR TITLE-ABS-KEY('minimally real change') OR TITLE-ABS-KEY('minimally real difference') OR TITLE-ABS-KEY('ceiling effect') OR TITLE-ABS-KEY('floor effect') OR TITLE-ABS-KEY('item response model') OR TITLE-ABS-KEY('irt') OR TITLE-ABS-KEY('rasch') OR TITLE-ABS-KEY('differential item functioning') OR TITLE-ABS-KEY('dif') OR TITLE-ABS-KEY('computer adaptive testing') OR TITLE-ABS-KEY('item bank') OR TITLE-ABSKEY('cross-cultural equivalence')

AND

TITLE-ABS-KEY ("chronic pain condition*" OR "central sensitivity syndrome*" OR "central pain syndrome*" OR "chronic primary pain condition*" OR "chronic pain*" OR "chronic overlapping pain condition*" OR "Functional somatic syndrome*" OR "chronic musculoskeletal pain*")

AND

TITLE-ABS-KEY ("Central sensitization*" OR "Central sensitisation*" OR "centralized pain*" OR "central pain*" or "nociplastic pain*" OR "pain sensitization*" OR "pain sensitivity*" OR "pain hypersensitization*" OR "central hyperexcitability*")

AND

TITLE-ABS-KEY ("Questionnaire*" OR "tool*" OR "scale*" OR "instrument*" or "measurement*" OR "index*" OR "central sensitization inventory*" OR "CSI*" OR "pain sensitivity questionnaire*" OR "Fibromyalgia survey questionnaire*" OR "Widespread Pain Index and Symptom Severity Scale*")

**Web of Science *1438 records***

TS=(("Central sensitization*" OR "Central sensitisation*" OR "centralized pain*" OR "central pain*" or "nociplastic pain*" OR "pain sensitization*" OR "pain sensitivity*" OR "pain hypersensitization*" OR "central hyperexcitability*")))

AND

TS=((chronic pain OR central pain syndrome OR chronic primary pain condition OR central sensitivity syndrome OR chronic pain condition OR chronic overlapping pain condition OR Functional somatic syndrome OR chronic musculoskeletal pain)))

AND

TS=((Questionnaire OR tool OR scale OR instrument OR measurement OR index OR central sensitization inventory OR CSI OR pain sensitivity questionnaire OR Fibromyalgia survey questionnaire OR Widespread Pain Index and Symptom Severity Scale))

AND

(TS="psychometric properties" OR TS="measurement properties" OR TS=psychometr* OR TS="outcome assessment" OR TS= "validation studies" OR TS="observer variation" OR TS=reproducib* OR TS=reliab* OR TS=unreliab* OR TS=valid* OR TS=coefficient OR TS=homogeneity OR TS=homogeneous OR TS="internal consistency" OR TS=(cronbach* AND (alpha OR alphas)) OR TS=(item AND (correlation* OR selection* OR reduction*)) OR TS=precision OR TS=imprecision OR TS=(precise values) OR TS=test-retest OR TS=(test AND retest) OR TS=(reliab* AND (test OR retest)) OR TS=stability OR TS=interrater OR TS=inter-rater OR TS=intrarater OR TS=intra-rater OR TS=intertester OR TS=inter-tester OR TS=intratester OR TS=intra-tester OR TS=interobserver OR TS=inter-observer OR TS=intraobserver OR TS=intra-observer OR TS=intertechnician OR TS=intertechnician OR TS=intratechnician OR TS=intra-technician OR TS=interexaminer OR TS=interexaminer OR TS=intraexaminer OR TS=intra-examiner OR TS=interassay OR TS=inter-assay OR TS=intraassay OR TS=intra-assay OR TS=interindividual OR TS=inter-individual OR TS=intraindividual OR TS=intra-individual OR TS=interparticipant OR TS=inter-participant OR TS=intraparticipant OR TS=intra-participant OR TS=kappa OR TS=kappa's OR TS=kappas OR TS=repeatab* OR TS=((replicab* OR repeated) AND (measure OR measures OR findings OR result OR results OR test OR tests)) OR TS=generaliza* OR TS=generalisa* OR TS=concordance OR TS=(intraclass AND correlation*) OR TS=discriminative OR TS=(known group) OR TS="factor analysis" OR TS="factor analyses" OR TS=dimension* OR TS=subscale* OR TS=(multitrait AND scaling AND (analysis OR analyses)) OR TS="item discriminant" OR TS="interscale correlation*" OR TS=(error OR errors) OR TS="individual variability" OR TS=(variability AND (analysis OR values)) OR TS=(uncertainty AND (measurement OR measuring)) OR TS="standard error" OR TS="of measurement" OR TS=sensitiv* OR TS=responsive* OR TS=((minimal OR minimally OR clinical OR clinically) AND (important OR significant OR detectable) AND (change OR difference)) OR TS=(small* AND (real OR detectable) AND (change OR difference)) OR TS="meaningful change" OR TS="minimal important change" OR TS="minimal important difference" OR TS="minimally important change" OR TS="minimally important difference" OR TS="minimal detectable change" OR TS="minimal detectable difference" OR TS="minimally detectable change” OR TS="minimally detectable difference" OR TS="minimal real change" OR TS="minimal real difference" OR TS="minimally real change" OR TS="minimally real difference" OR TS="ceiling effect" OR TS="floor effect" OR TS="Item response model" OR TS=IRT OR TS=Rasch OR TS="Differential item functioning" OR TS=DIF OR TS="computer adaptive testing" OR TS="item bank" OR TS="cross-cultural equivalence")

**CINAHL *912 records***

(TI psychometr* OR TI observer variation OR TI reproducib* OR TI reliab* OR TI unreliab* OR TI valid* OR TI coefficient OR TI homogeneity OR TI homogeneous OR TI "internal consistency" OR AB psychometr* OR AB observer variation OR AB reproducib* OR AB reliab* OR AB unreliab* OR AB valid* OR AB coefficient OR AB homogeneity OR AB homogeneous OR AB "internal consistency" OR (TI cronbach* OR AB cronbach* AND (TI alpha OR AB alpha OR TI alphas OR AB alphas)) OR (TI item OR AB item AND (TI correlation* OR AB correlation* OR TI selection* OR AB selection* OR TI reduction* OR AB reduction*)) OR TI agreement OR TI precision OR TI imprecision OR TI "precise values" OR TI test-retest OR AB agreement OR AB precision OR AB imprecision OR AB "precise values" OR AB test-retest OR (TI test OR AB test AND TI retest OR AB retest) OR (TI reliab* OR AB reliab* AND (TI test OR AB test OR TI retest or AB retest)) OR TI stability OR TI interrater OR TI interrater OR TI intrarater OR TI intra-rater OR TI intertester OR TI inter-tester OR TI intratester OR TI intra-tester OR TI interobserver OR TI inter-observer OR TI intraobserver OR TI intra-observer OR TI intertechnician OR TI inter-technician OR TI intratechnician OR TI intra-technician OR TI interexaminer OR TI inter-examiner OR TI intraexaminer OR TI intraexaminer OR TI interassay OR TI inter-assay OR TI intraassay OR TI intra-assay OR TI interindividual OR TI inter-individual OR TI intraindividual OR TI intra-individual OR TI interparticipant OR TI inter-participant OR TI intraparticipant OR TI intra-participant OR TI kappa OR TI kappa's OR TI kappas OR TI repeatab* OR AB stability OR AB interrater OR AB inter-rater OR AB intrarater OR AB intra-rater OR AB intertester OR AB inter-tester OR AB intratester OR AB intra-tester OR AB interobserver OR AB inter-observer OR AB intraobserver OR AB intra-observer OR AB intertechnician OR AB inter-technician OR AB intratechnician OR AB intra-technician OR AB interexaminer OR AB inter-examiner OR AB intraexaminer OR AB intra-examiner OR AB interassay OR AB inter-assay OR AB intraassay OR AB intra-assay OR AB interindividual OR AB inter-individual OR AB intraindividual OR AB intra-individual OR AB interparticipant OR AB inter-participant OR AB intraparticipant OR AB intra-participant OR AB kappa OR AB kappa's OR AB kappas OR AB repeatab* OR ((TI replicab* OR AB replicab* OR TI repeated OR AB repeated) AND (TI measure OR AB measure OR TI measures OR AB measures OR TI findings OR AB findings OR TI result OR AB result OR TI results OR AB results OR TI test OR AB test OR TI tests OR AB tests)) OR TI generaliza* OR TI generalisa* OR TI concordance OR AB generaliza* OR AB generalisa* OR AB concordance OR (TI intraclass OR AB intraclass AND TI correlation* or AB correlation*) OR TI discriminative OR TI "known group" OR TI factor analysis OR TI factor analyses OR TI dimension* OR TI subscale* OR AB discriminative OR AB "known group" OR AB factor analysis OR AB factor analyses OR AB dimension* OR AB subscale* OR (TI multitrait OR AB multitrait AND TI scaling OR AB scaling AND (TI analysis OR AB analysis OR TI analyses OR AB analyses)) OR TI item discriminant OR TI interscale correlation* OR TI error OR TI errors OR TI "individual variability" OR AB item discriminant OR AB interscale correlation* OR AB error OR AB errors OR AB "individual variability” OR (TI variability OR AB variability AND (TI analysis OR AB analysis OR TI values OR AB values)) OR (TI uncertainty OR AB uncertainty AND (TI measurement OR AB measurement OR TI measuring OR AB measuring)) OR TI "standard error of measurement" OR TI sensitiv* OR TI responsive* OR AB "standard error of measurement" OR AB sensitiv* OR AB responsive* OR ((TI minimal OR TI minimally OR TI clinical OR TI clinically OR AB minimal OR AB minimally OR AB clinical OR AB clinically) AND (TI important OR TI significant OR TI detectable OR AB important OR AB significant OR AB detectable) AND (TI change OR AB change OR TI difference OR AB difference)) OR (TI small* OR AB small* AND (TI real OR AB real OR TI detectable OR AB detectable) AND (TI change OR AB change OR TI difference OR AB difference)) OR TI meaningful change OR TI "ceiling effect" OR TI "floor effect" OR TI "Item response model" OR TI IRT OR TI Rasch OR TI “Differential item functioning" OR TI DIF OR TI "computer adaptive testing" OR TI “item bank” OR TI "cross-cultural equivalence" OR TI outcome assessment OR AB meaningful change OR AB "ceiling effect" OR AB "floor effect" OR AB "Item response model" OR AB IRT OR AB Rasch OR AB "Differential item functioning" OR AB DIF OR AB "computer adaptive testing" OR AB "item bank" OR AB "cross-cultural equivalence" OR AB outcome assessment)

AND

TX(Central sensitization OR centralized pain OR central pain OR nociplastic pain OR pain sensitization OR pain sensitivity OR pain hypersensitization OR central hyperexcitability

AND

TX (chronic pain OR central pain syndrome OR chronic primary pain condition OR central sensitivity syndrome OR chronic pain condition OR chronic overlapping pain condition OR Functional somatic syndrome OR chronic musculoskeletal pain)

AND

TX (Questionnaire OR tool OR scale OR instrument OR measurement OR index OR central sensitization inventory OR CSI OR pain sensitivity questionnaire OR Fibromyalgia survey questionnaire OR Widespread Pain Index and Symptom Severity Scale)
