# Supplementary material for: Psychometric properties of patient-reported outcome measures in chronic pain conditions with central sensitization- a systematic review and meta-analysis
Source: J Patient Rep Outcomes. 2025 Jul 11;9:87. doi: 10.1186/s41687-025-00919-9 (PMC12254461; doi:10.1186/s41687-025-00919-9)
Supplement: Supplementary file 2 — Supplementary Material 2 [file 41687_2025_919_MOESM2_ESM.docx]

**Appendix B: “Do file”- Codes for meta-analysis**

generate z = atanh(r) // r-to-z = inverse hyperbolic tangent

generate sez = sqrt(1/(n - 3))

*admetan z sez

metan z sez, label(namevar = study)

display _newline ///

" Pooled estimate of r = " tanh(r(eff)) _newline ///

"Lower Limit of 95% CI = " tanh(r(eff) - (1.96 * r(se_eff))) _newline ///

"Upper Limit of 95% CI = " tanh(r(eff) + (1.96 * r(se_eff)))

// Prepare data for -forestplot-

generate _USE = 1

// Generate CI's for r

generate lb = tanh(_LCI)

generate ub = tanh(_UCI)

// Generate CI's for r

gen effect = tanh(r(eff))

generate lb = tanh(_LCI)

generate ub = tanh(_UCI)

label var n "Sample size"

*Restricted maximum liklihood method

metan r lb ub, label(namevar = study) random(reml) ///

xlab(0(0.2)1) ///

xscale(range(0 1)) ///

xlabel(0(0.2)1, format(%3.1f)) ///

overall
