# Supplementary material for: Psychometric properties of patient-reported outcome measures in chronic pain conditions with central sensitization- a systematic review and meta-analysis
Source: J Patient Rep Outcomes. 2025 Jul 11;9:87. doi: 10.1186/s41687-025-00919-9 (PMC12254461; doi:10.1186/s41687-025-00919-9)
Supplement: Supplementary file 3 — Supplementary Material 3 [file 41687_2025_919_MOESM3_ESM.docx]

**Appendix C: COSMIN ratings on methodological quality, results and overall rating per measurement property**

| **Table 6 COSMIN ratings on methodological quality, results, and overall rating per measurement property** | | | | | | | | | |
| --- | --- | --- | --- | --- | --- | --- | --- | --- | --- |
| **COSMIN measurement properties** | **CSI [30-66]** | | | **PSQ 46,67-73]** | | | **FSQ [74-82]** | | |
|  | **Studies (Meth Qual Rating)** | **Results (Rating)** | **Summary of Results (Overall Rating)** | **Studies (Meth Qual Rating)** | **Results (Rating)** | **Summary of Results (Overall Rating)** | **Studies (Meth Qual Rating)** | **Results (Rating)** | **Summary of Results (Overall Rating)** |
|  | **V/A/D/I*** | **+/−/? **** | **+/−/±/?**** | **V/A/D/I*** | **+/−/? **** | **+/−/±/?**** | **V/A/D/I*** | **+/−/? **** | **+/−/±/?**** |
| **Content validity** | Bid 2016 (D) | Relevance: (+) Comprehensiveness: (+) Comprehensibility: (+) | **Content validity: (+)** | Ibancos-Losada 2021 (D) | Relevance: (+) Comprehensiveness: (+) Comprehensibility: (+) | **Content validity: (+)** | Carrillo-de-la-Peña 2015 (D) | Relevance: (+) Comprehensiveness: (+) Comprehensibility: (+) | **Content validity: (+)** |
|  | Noorollahzadeh2021 (D) | Relevance: (+) Comprehensiveness: (+) Comprehensibility: (+) |  | Latka 2019 (D) | Relevance: (+) Comprehensiveness: (+) Comprehensibility: (+) |  | Aguirre Cárdenas 2021 (D) | Relevance: (+) Comprehensiveness: (+) Comprehensibility: (+) |  |
|  | Liang 2022 (D) | Relevance: (+) Comprehensiveness: (+) Comprehensibility: (+) |  | Kim 2014 (D) | Relevance: (+) Comprehensiveness: (+) Comprehensibility: (+) |  |  |  |  |
|  | Wiangkham 2022 (D) | Relevance: (+) Comprehensiveness: (+) Comprehensibility: (+) |  |  |  |  |  |  |  |
|  | Sharma 2020 (D) | Relevance: (+) Comprehensiveness: (+) Comprehensibility: (+) |  |  |  |  |  |  |  |
|  | Caumo 2017 (D) | Relevance: (+) Comprehensiveness: (+) Comprehensibility: (+) |  |  |  |  |  |  |  |
|  | Knezevic 2018 (D) | Relevance: (+) Comprehensiveness: (+) Comprehensibility: (+) |  |  |  |  |  |  |  |
|  | Mikkonen 2021 (D) | Relevance: (+) Comprehensiveness: (+) Comprehensibility: (+) |  |  |  |  |  |  |  |
|  | Chiarotto 2018 (D) | Relevance: (+) Comprehensiveness: (+) Comprehensibility: (+) |  |  |  |  |  |  |  |
|  | Madi 2021 (D) | Relevance: (+) Comprehensiveness: (+) Comprehensibility: (+) |  |  |  |  |  |  |  |
|  | Klute 2021 (A) | Relevance: (+) Comprehensiveness: (+) Comprehensibility: (+) |  |  |  |  |  |  |  |
|  | Holm 2021 (D) | Relevance: (+) Comprehensiveness: (+) Comprehensibility: (+) |  |  |  |  |  |  |  |

* V = very good, A = adequate, D = doubtful, I = inadequate; ** + = sufficient, - = insufficient, ± = inconsistent, ?= indeterminate

| **Table 6 COSMIN ratings on methodological quality, results, and overall rating per measurement property *cont.*** | | | | | | | | | |  |
| --- | --- | --- | --- | --- | --- | --- | --- | --- | --- | --- |
| **COSMIN measurement properties** | | **CSI [30-66]** | | | **PSQ 46,67-73]** | | | **FSQ [74-82]** | | |
|  |  | **Studies (Meth Qual Rating)** | **Results (Rating)** | **Summary of Results (Overall Rating)** | **Studies (Meth Qual Rating)** | **Results (Rating)** | **Summary of Results (Overall Rating)** | **Studies (Meth Qual Rating)** | **Results (Rating)** | **Summary of Results (Overall Rating)** |
|  |  | **V/A/D/I*** | **+/−/? **** | **+/−/±/?**** | **V/A/D/I*** | **+/−/? **** | **+/−/±/?**** | **V/A/D/I*** | **+/−/? **** | **+/−/±/?**** |
| **Structural validity** | | Mayer 2012 (D) | EFA → factor 1 =0.48–0.91; factor 2 =0.47–0.82; factor 3= 0.55–0.73; factor 4= 0.41- 0.71 (+) | **Acceptable factor loading (+)** | Ibancos-Losada 2021 (I) | EFA → The factorial analysis explained 69% of the variance. PSQ-moderate =0.627-0.783; PSQ-minor= 0.609-0.847 (+) | **2 factors with acceptable factor loading (+)** | NA | NA | NA |
|  |  | Noorollahzadeh  2021 (V) | CFA → Root Mean Square Error of Approximation (RMSEA)=0.049 (+) |  | Latka 2019 (A) | EFA → The factorial analysis revealed 70.69% of the total variance. (+) |  |  |  |  |
|  |  | Liang 2022 (V) | CFA → factor 1=0.45–0.84; factor 2 =0.43–0.80; factor 3= 0.42–0.73; factor 4= 0.44- 0.64. Fit Indices were not reported. (+) |  | Kim 2014 (I) | PSQ-moderate (factor 1)= 0.510-0.782, PSQ-minor (factor 2)= 0.529-0.871 (+) |  |  |  |  |
|  |  | Roby 2022 (V) | IRT → No violation of unidimensionality, local independence (Following iterative creation of subtests ), monotonicity and the chi-square value for item-trait interaction (X2 (80)=99.1, P=0.071) was non-significant which indicates fit to the Rasch model. (+) |  |  |  |  |  |  |  |
|  |  | Bakhtadze 2022 (A) | EFA → one-factors model: 0.263-0.715 (8 out of 25 items had factor loading < 0.40). (-) |  |  |  |  |  |  |  |
|  |  | Wiangkham 2022 (V) | CFA → One-factor model: CFI=0.89, TLI=0.88, RMSEA=0.09; Correlated 4-factor model: CFI= 0.90, TLI= 0.89, RMSEA= 0.08 ; Bifactor model: CFI= 0.93, TLI= 0.92, RMSEA= 0.070 (-) |  |  |  |  |  |  |  |
|  |  | Bakhtadze 2021 (A) | EFA→ 6 factor model: factor 1=0.48-0.75; factor 2= 0.49-0.68; factor 3= 0.55-.66; factor 4= 0.46-0.79; factor 5= 0.49-0.68; factor 6= 0.62-0.64 (+) |  |  |  |  |  |  |  |
|  |  | Düzce Keleş 2021 (A) | EFA→ The first 7, which was greater than an eigenvalue of the scale of 7 described 61.1% of the variance. There was a clear levelling off in the scree plot after the first factor. (+) |  |  |  |  |  |  |  |
|  |  | Tanaka 2017 (A) | EFA→ 5 factors model factor 1= 0.40-1.03; factor 2= 0.41-0.75; factor 3= 0.49-0.75; factor 4= 0.45-0.57; factor 5= 0.42-0.54. The factor loading of 7 items: <0.40. (-) |  |  |  |  |  |  |  |
|  |  | Caumo 2017 (V) | CFA → factor 1 = 0.42-0.88; factor 2 = 0.5-.78; factor 3= 0.5-0.64; factor 4= 0.47-0.64 (+) |  |  |  |  |  |  |  |

| **Table 6 COSMIN ratings on methodological quality, results, and overall rating per measurement property *cont.*** | | | | | | | | | |
| --- | --- | --- | --- | --- | --- | --- | --- | --- | --- |
| **COSMIN measurement properties** | **CSI [30-66]** | | | **PSQ 46,67-73]** | | | **FSQ [74-82]** | | |
|  | **Studies (Meth Qual Rating)** | **Results (Rating)** | **Summary of Results (Overall Rating)** | **Studies (Meth Qual Rating)** | **Results (Rating)** | **Summary of Results (Overall Rating)** | **Studies (Meth Qual Rating)** | **Results (Rating)** | **Summary of Results (Overall Rating)** |
|  | **V/A/D/I*** | **+/−/? **** | **+/−/±/?**** | **V/A/D/I*** | **+/−/? **** | **+/−/±/?**** | **V/A/D/I*** | **+/−/? **** | **+/−/±/?**** |
| **Structural validity** | Kregel 2016 (V) | EFA→ factor 1 = 0.41-0.71; factor 2 = 0.41-.77; factor 3= 0.44-0.72; factor 4= 0.45-0.83 CFA→ CFI: 0.97; NFI: 0.95 NNFI:0.97; RMSEA: 0.065 (+) |  |  |  |  |  |  |  |
|  | Feng 2022 (A) | EFA→ factor 1= 0.44-0.78; factor 2=0.495-0.78; factor 3=0.77-0.80; factor 4 =0.46-0.56; factor 5= 0.48-0.59 (10 items not loaded on the factor) (-) |  |  |  |  |  |  |  |
|  | Cuesta-Vargas 2016 (A) | EFA→ The percentage of total variance explained by the one factor = 25.9% (-) |  |  |  |  |  |  |  |
|  | Knezevic 2018 (V) | CFA→ factor 1= 0.41-0.78; factor 2=0.56-0.81; factor 3= 0.51-0.64; factor 4=0.63-0.77; RMSEA = 0.07; CFI = 0.93; TLI = 0.93. (-) |  |  |  |  |  |  |  |
|  | Mikkonen 2021 (A) | EFA→ One factor model: 0.30-0.64; The percentage of total variance explained by factor 1= 28.1% (Eigenvalue 7.026) and factor 2= 34.9% (1.706) (-) |  |  |  |  |  |  |  |
|  | Kosińska 2021 (A) | EFA→ Factor 1= 0.406-0.787; factor 2= 0.517-0.801; factor 3= 0.465-0.716; factor 4= 0.349-0.758; CFA→ RMSEA=0.07 (+) |  |  |  |  |  |  |  |
|  | Kim et al., 2020 (A) | EFA→ factor 1= 0.462-0.836; factor 2= 0.403-0.768; factor 3= 0.652-0.755; factor 4= 0.411-0.863; factor 5= 0.436-0.883; factor 6= 0.512-0.872. (+) |  |  |  |  |  |  |  |
|  | Chiarotto 2018 (A) | EFA→ EFA revealed a first eigenvalue accounted for 26% of the total variance, and the ratio of the first to the second eigenvalue was 3.8. Factor loading = -0.09-0.61. (-) |  |  |  |  |  |  |  |
|  | Madi 2021 (A) | EFA→ factor 1= 0.503-0.754; factor 2= 0.504-0.747; factor 3= 0.467-0.621; factor 4=0.400- 0.724 (+) |  |  |  |  |  |  |  |
|  | Klute 2021 (V) | CFA→ 4-factor model; TLI = 0.99; RMSEA = 0.06; x2 (269) = 553.09, p < 0.001. 1-factor model; TLI = 0.98; RMSEA = 0.08; χ2(275) = 756.39, p < 0.001. Bifactor model; TLI = 0.99; RMSEA = 0.05; x2 (250) = 430.6, p < 0.001. (+) |  |  |  |  |  |  |  |

| **Table 6 COSMIN ratings on methodological quality, results, and overall rating per measurement property *cont.*** | | | | | | | | | |
| --- | --- | --- | --- | --- | --- | --- | --- | --- | --- |
| **COSMIN measurement properties** | **CSI [30-66]** | | | **PSQ 46,67-73]** | | | **FSQ [74-82]** | | |
|  | **Studies (Meth Qual Rating)** | **Results (Rating)** | **Summary of Results (Overall Rating)** | **Studies (Meth Qual Rating)** | **Results (Rating)** | **Summary of Results (Overall Rating)** | **Studies (Meth Qual Rating)** | **Results (Rating)** | **Summary of Results (Overall Rating)** |
|  | **V/A/D/I*** | **+/−/? **** | **+/−/±/?**** | **V/A/D/I*** | **+/−/? **** | **+/−/±/?**** | **V/A/D/I*** | **+/−/? **** | **+/−/±/?**** |
| **Internal consistency** | Mayer 2012 (V) | Cronbach’s alpha (total): 0.88 (+) | **Cronbach’s α = 0.87–0.99 (+)** | Ibancos-Losada 2021 (V) | Cronbach’s α PSQ-total = 0.95; PSQ-moderate = 0.91; and PSQ-minor = 0.92 (+) | **Cronbach’s α = 0.87–0.96 (+)** | Carrillo-de-la-Peña 2015 (V) | Cronbach’s α: FSQ total (PSD) = 0.85 (?) | **Cronbach’s α = 0.71–0.94 (?)** |
|  | Bid 2016 (I) | Cronbach’s alpha (total): 0.91 (?) |  | Latka 2019 (V) | Cronbach’s α PSQ-total = 0.96 (+) |  | Häuser 2012 (V) | Cronbach’s α: FS (WPI+SSS) = 0.71 (?) |  |
|  | Noorollahzadeh  2021 (V) | Cronbach’s α = 0.87 (+) |  | Kim 2014 (V) | Cronbach’s α PSQ-total = 0.93; PSQ-moderate = 0.88; and PSQ-minor = 0.87 (+) |  | Fors et al 2020 (V) | Cronbach’s α: FS (WPI+SSS) = 0.90 (?) |  |
|  | Liang 2022 (V) | Cronbach’s α(total)= 0.88 (+) |  |  |  |  | Jiao 2023 (V) | Cronbach’s α: FS (WPI+SSS) = 0.82 (?) |  |
|  | Roby 2022 (V) | Cronbach’s alpha (total)= 0.89 and Person Separation Index (PSI)= 0.91.after rescoring the thresholds. (+) |  |  |  |  | Kang 2019 (I) | Cronbach’s α (total) = 0.94 (?) |  |
|  | Bakhtadze 2022 (V) | Cronbach’s α (total) = 0.88 (?) |  |  |  |  | Aguirre Cárdenas 2021 (V) | Cronbach’s α: FSQ (total) at T1= 0.91 and T2= 0.78 (?) |  |
|  | Wiangkham 2022 (V) | Cronbach’s α (total) = 0.91 (?) |  |  |  |  | Bidari 2015 (V) | Cronbach’s α: FSQ total (WPI+SSS=PSD) = 0.81 (?) |  |
|  | Bakhtadze 2021 (V) | Cronbach’s α (total) =0.89 (+) |  |  |  |  |  |  |  |
|  | Sharma 2020 (I) | Cronbach’s α (total) = 0.87 (?) |  |  |  |  |  |  |  |
|  | Düzce Keleş 2021 (V) | Cronbach’s α (total) = 0.92 (+) |  |  |  |  |  |  |  |
|  | Tanaka 2017 (V) | Cronbach’s α (total) =0.89 (?) |  |  |  |  |  |  |  |
|  | Caumo 2017 (V) | Cronbach’s α (total) = 0.91 (+) |  |  |  |  |  |  |  |
|  | Kregel 2016 (V) | Cronbach’s α (total) = 0.91 (+) |  |  |  |  |  |  |  |
|  | Feng 2022 (V) | Cronbach’s α (total) = 0.89 (?) |  |  |  |  |  |  |  |
|  | Cuesta-Vargas 2016 (V) | Cronbach’s α (total) = 0.87 (?) |  |  |  |  |  |  |  |
|  | Knezevic 2018 (V) | Cronbach’s α (total) = 0.91 (?) |  |  |  |  |  |  |  |
|  | Mikkonen 2021 (V) | Cronbach’s α (total) = 0.88 (?) |  |  |  |  |  |  |  |
|  | Kosińska 2021 (V) | Cronbach’s α (total) = 0.93 (+) |  |  |  |  |  |  |  |
|  | Bilika 2020 (V) | Cronbach’s α (total) = 0.99 (?) |  |  |  |  |  |  |  |
|  | Kim 2020 (V) | Cronbach’s α (total) = 0.94 (+) |  |  |  |  |  |  |  |
|  | Chiarotto 2018 (V) | Cronbach’s α (total) = 0.87 (?) |  |  |  |  |  |  |  |
|  | Madi 2021 (V) | Cronbach’s α (total) = 0.88 (+) |  |  |  |  |  |  |  |
|  | Klute 2021 (V) | Cronbach’s α (total) = 0.93 (+) |  |  |  |  |  |  |  |

| **Table 6 COSMIN ratings on methodological quality, results, and overall rating per measurement property *cont.*** | | | | | | | | | |
| --- | --- | --- | --- | --- | --- | --- | --- | --- | --- |
| **COSMIN measurement properties** | **CSI [30-66]** | | | **PSQ 46,67-73]** | | | **FSQ [74-82]** | | |
|  | **Studies (Meth Qual Rating)** | **Results (Rating)** | **Summary of Results (Overall Rating)** | **Studies (Meth Qual Rating)** | **Results (Rating)** | **Summary of Results (Overall Rating)** | **Studies (Meth Qual Rating)** | **Results (Rating)** | **Summary of Results (Overall Rating)** |
|  | **V/A/D/I*** | **+/−/? **** | **+/−/±/?**** | **V/A/D/I*** | **+/−/? **** | **+/−/±/?**** | **V/A/D/I*** | **+/−/? **** | **+/−/±/?**** |
| Cross cultural validity | NA | NA | NA | NA | NA | NA | NA | NA | NA |
| **Reliability** | Mayer 2012 (D) | ICC or weighted kappa not reported. Pearson correlation (test–retest correlation) for the total score = 0.817 (?) | **Test-retest ICC = 0.85–0.99 (+)** | Ruscheweyh 2012 (I) | Test-retest: ICC (PSQ-total) = 0.72; ICC (PSQ-minor)= 0.71. (+) | **Test-retest ICC = 0.71–0.93(+)** | Fors et al 2020 (A) | Test-retest: ICC (FS =WPI+SSS) = 0.86 (+) | **Test-retest ICC = 0.79–0.86 (+)** |
|  | Bid 2016 (A) | Test-retest: ICC (total) = 0.971 (+) |  | Latka 2019 (I) | Test-retest: ICC (PSQ-total) = 0.92; ICC (PSQ-moderate)= 0.87; ICC (PSQ-minor)= 0.91. (+) |  | Jiao 2023 (D) | ICC or weighted kappa not reported. Spearman’s correlation analysis for the FS scale, its subscales r = 0.53 to 0.82 (?) |  |
|  | Noorollahzadeh  2021 (A) | Test-retest: ICC (total) =0.934 (+) |  | Kim 2014 (I) | Test-retest: ICC (PSQ-total) = 0.78; ICC (PSQ-moderate)= 0.79; ICC (PSQ-minor)= 0.75. (+) |  | Kang 2019 (D) | ICC or weighted kappa not reported. Spearman’s correlation analysis ranged from 0.616 to 0.910 (?) |  |
|  | Liang 2022 (A) | Test-retest: ICC (total) =0.934 (+) |  |  |  |  | Aguirre Cárdenas 2021 (I) | Test-retest: ICC ( FSQ total) = 0.79 (+) |  |
|  | Bakhtadze 2022 (I) | Test-retest: ICC (total) =0.91 (+) |  |  |  |  |  |  |  |
|  | Wiangkham 2022 (I) | Test-retest: ICC (total) = 0.90 (+) |  |  |  |  |  |  |  |
|  | Bakhtadze 2021 (I) | Test-retest: ICC (total) = 0.89 (+) |  |  |  |  |  |  |  |
|  | Sharma 2020 (I) | Test-retest: ICC (total) = 0.98 (+) |  |  |  |  |  |  |  |
|  | Düzce Keleş 2021 (A) | Test-retest: ICC (total) = 0.93 (+) |  |  |  |  |  |  |  |
|  | Tanaka 2017 (A) | Test-retest: ICC (total) = 0.85 (+) |  |  |  |  |  |  |  |
|  | Caumo 2017 (A) | Test-retest: ICC (total) = 0.91 (+) |  |  |  |  |  |  |  |
|  | Kregel 2016 (I) | Test-retest: ICC (total = 0.88 (+) |  |  |  |  |  |  |  |
|  | Feng 2022 (I) | Test-retest: ICC (total) = 0.932 (+) |  |  |  |  |  |  |  |
|  | Cuesta-Vargas 2016 (A) | Test-retest: ICC (total) = 0.91 (+) |  |  |  |  |  |  |  |
|  | Knezevic 2018 (V) | Test-retest: ICC (total) = 0.947 (+) |  |  |  |  |  |  |  |
|  | Mikkonen 2021 (A) | Test-retest: ICC (total) = 0.933 (+) |  |  |  |  |  |  |  |
|  | Kosińska 2021 (A) | Test-retest: ICC (total) = 0.96 (+) |  |  |  |  |  |  |  |
|  | Bilika 2020 (A) | Test-retest: ICC (total) = 0.991 (+) |  |  |  |  |  |  |  |
|  | Kim 2020 (A) | Test-retest: ICC (total) = 0.888 (+) |  |  |  |  |  |  |  |
|  | Madi 2021 (A) | Test-retest: ICC (total) = 0.94 (+) |  |  |  |  |  |  |  |
|  | Klute 2021 (A) | Test-retest: ICC (total) = 0.917(+) |  |  |  |  |  |  |  |

| **Table 6 COSMIN ratings on methodological quality, results, and overall rating per measurement property *cont.*** | | | | | | | | | |
| --- | --- | --- | --- | --- | --- | --- | --- | --- | --- |
| **COSMIN measurement properties** | **CSI [30-66]** | | | **PSQ 46,67-73]** | | | **FSQ [74-82]** | | |
|  | **Studies (Meth Qual Rating)** | **Results (Rating)** | **Summary of Results (Overall Rating)** | **Studies (Meth Qual Rating)** | **Results (Rating)** | **Summary of Results (Overall Rating)** | **Studies (Meth Qual Rating)** | **Results (Rating)** | **Summary of Results (Overall Rating)** |
|  | **V/A/D/I*** | **+/−/? **** | **+/−/±/?**** | **V/A/D/I*** | **+/−/? **** | **+/−/±/?**** | **V/A/D/I*** | **+/−/? **** | **+/−/±/?**** |
| **Measurement error** | Bid 2016 (A) | Variability → SEM= 1.84; Smallest detectable change (SDC) / MDC = 5.09 (?) | **SEM = 0.31– 4.14; SDC( MDC) = 0.86 –11.5 (MIC not determined) (?)** | NA | NA | NA | NA | NA | NA |
|  | Bakhtadze 2022 (I) | Minimal detectable changes (MDC)= 10 (?) |  |  |  |  |  |  |  |
|  | Wiangkham 2022 (I) | Variability → SEM=2.33; Minimal detectable changes (MDC)= 6.47 (?) |  |  |  |  |  |  |  |
|  | Bakhtadze 2021 (I) | Minimal detectable changes (MDC)= 10 (?) |  |  |  |  |  |  |  |
|  | Sharma 2020 (I) | Variability → SEM= 0.31; Smallest detectable change (SDC)= 0.86 (?) |  |  |  |  |  |  |  |
|  | Cuesta-Vargas 2016 (A) | Variability → SEM= 2.52 ; minimum detectable change (MDC90)= 7.83% (?) |  |  |  |  |  |  |  |
|  | Knezevic 2018 (V) | Variability → SEM= 3.16% ; minimum detectable change (MDC90)= 8.12% (?) |  |  |  |  |  |  |  |
|  | Mikkonen 2021 (A) | Variability → SEM= 0.43 (?) |  |  |  |  |  |  |  |
|  | Kosińska 2021 (A) | Variability → SEM= 0.99; minimum detectable change (MDC90)= 2.31% (?) |  |  |  |  |  |  |  |
|  | Bilika 2020 (A) | Variability → SEM= 2.1 (?) |  |  |  |  |  |  |  |
|  | Madi 2021 (A) | Variability → SEM= 3.45; minimum detectable change (MDC)= 9.657 (?) |  |  |  |  |  |  |  |
|  | Klute 2021 (A) | Variability → SEM= 4.14; Smallest detectable change (SDC)= ± 11.49 (?) |  |  |  |  |  |  |  |
| **Criterion validity** | NA | NA | NA | NA | NA | NA | NA | NA | NA |

| **Table 6 COSMIN ratings on methodological quality, results, and overall rating per measurement property *cont.*** | | | | | | | | | |
| --- | --- | --- | --- | --- | --- | --- | --- | --- | --- |
| **COSMIN measurement properties** | **CSI [30-66]** | | | **PSQ 46,67-73]** | | | **FSQ [74-82]** | | |
|  | **Studies (Meth Qual Rating)** | **Results (Rating)** | **Summary of Results (Overall Rating)** | **Studies (Meth Qual Rating)** | **Results (Rating)** | **Summary of Results (Overall Rating)** | **Studies (Meth Qual Rating)** | **Results (Rating)** | **Summary of Results (Overall Rating)** |
|  | **V/A/D/I*** | **+/−/? **** | **+/−/±/?**** | **V/A/D/I*** | **+/−/? **** | **+/−/±/?**** | **V/A/D/I*** | **+/−/? **** | **+/−/±/?**** |
| **Hypothesis testing (construct validity)** | Mayer 2012 (V) | Discriminative validity → There was a significant difference in the total score of CSI (≥ 10 points higher) between patient subgroups compared to the Control group, p<0.001. The fibromyalgia group scored significantly the highest than other patient subgroups. (1+) | **Results in line with 106 hypotheses but not with 22 hypotheses (+)** | Ruscheweyh 2012 (A) | Convergent validity→ PSQ (total) and QST r = -0.47, P < .001; PSQ (minor) and QST r = -0.52, P < .001; PSQ(total) and QST r = 0.76, P < .001; PSQ (minor) and QST r = 0.71, P < .001; PSQ-minor and BDI r= 0.09, P> 0.05; PSQ-minor and PCS r= 0.25, P < 0.01; PSQ-minor and STAI r=0.19, P < 0.05. Discriminative validity → PSQ-total scores and PSQ-minor scores were significantly higher in chronic pain patients (but ˂ 10 points higher) than the control group, P < .001. (3+, 5-) | **Results in line with 38 hypotheses but not with 37 hypotheses (-)** | Carrillo-de-la-Peña 2015 (A) | Convergent validity→ FSQ total (PSD) and PHQ-15 r= 0.76, p < .001; FSQ (subscale) and PHQ-15 r= 0.73- 0.67, p < .001; FSQ total (PSD) and PSQI r= 0.45, p < .001; FSQ (subscale) and PSQI r= 0.32-0.58, p < .001; FSQ total (PSD) and PHQ-9 r= 0.62, p < .001; FSQ (subscale) and FIQ (symptom) r= 0.49-0.72, p < .001; FSQ total (PSD) and BDI r= 0.51, p < .001; FSQ (subscale) and BDI r= 0.41- 0.56, p < .001; FSQ total (PSD) and FIQ r= 0.72, p < .001; FSQ (subscale) and FIQ r=0.57-0.82, p < .001; FSQ total (PSD) and SF-12 r= -0.57, p < .001; FSQ (subscale) and SF-12 r= -0.42- to -0.71, p < .001. (12+) | **Results in line with 51 hypotheses but not with 15 hypotheses (+)** |
|  | Noorollahzadeh 2021 (V) | Discriminative validity → The patient subgroups showed the highest CSI score (≥ 10 points) than the control group as expected, p<0.001. (1+) |  | Sellers 2013 (A) | Convergent validity→ PSQ (total) and PCS r=0.32, P < 0.001; PSQ-minor and PCS r= 0.33 P < 0.001; PSQ- moderate and PCS r= 0.33 P < 0.001; PSQ(total) and HADS (depression, anxiety) r= 0.05-0.14, P>0.05; PSQ(subscales) and HADS (depression, anxiety) r= 0.03-0.13, P>0.05; PSQ-total and BPI (Pain, interference) r= 0.15-0.25, P>0.05; PSQ (subscales) and BPI (Pain, interference) r= 0.11-0.24, P>0.05; PSQ-total and RMQ r= 0.21, P>0.05; PSQ (subscales) and RMQ r=0.17- 0.22, P>0.05; PSQ(total) and VAS1 r= 0.23, P < 0.05; PSQ (subscale) and VAS1 r= 0.19-0.26, P < 0.05; PSQ(total) and VAS2 r= 0.33, P < 0.001;PSQ (subscale) and VAS2 r= 0.29-0.34, P < 0.001. (4+, 10-) |  | Häuser 2012 (A) | Convergent validity→ SSS and PHQ-4 r= 0.56, p˂ 0.0001; FS (WPI+SSS) and   PHQ-4 r= 0.48, p˂ 0.001. (2+) |  |

| **Table 6 COSMIN ratings on methodological quality, results, and overall rating per measurement property *cont.*** | | | | | | | | | |
| --- | --- | --- | --- | --- | --- | --- | --- | --- | --- |
| **COSMIN measurement properties** | **CSI [30-66]** | | | **PSQ 46,67-73]** | | | **FSQ [74-82]** | | |
|  | **Studies (Meth Qual Rating)** | **Results (Rating)** | **Summary of Results (Overall Rating)** | **Studies (Meth Qual Rating)** | **Results (Rating)** | **Summary of Results (Overall Rating)** | **Studies (Meth Qual Rating)** | **Results (Rating)** | **Summary of Results (Overall Rating)** |
|  | **V/A/D/I*** | **+/−/? **** | **+/−/±/?**** | **V/A/D/I*** | **+/−/? **** | **+/−/±/?**** | **V/A/D/I*** | **+/−/? **** | **+/−/±/?**** |
| **Hypothesis testing (construct validity)** | Liang 2022 (A) | Convergent validity → CSI and PCS (total) r=0.709; CSI and PCS subscales (rumination, magnification and helplessness) r=0.630–0.695; CSI and BPI (total) r=0.773; CSI and BPI (mean item score ) r=0.773. Discriminant validity → The patient subgroups showed the highest CSI score (≥ 10 points) than control group as expected, p<0.001. (5+) |  | Ibancos-Losada 2021 (V) | Convergent validity→ PSQ (total) and HADS (anxiety, depression) r= 0.45-0.49, p < 0.01; PSQ (subscales) and HADS (anxiety, depression) r=0.37-0.52, p < 0.01; PSQ (total) and PCS r= 0.58, p < 0.01; PSQ (subscales) and PCS r=0.50-0.60, p < 0.01; PSQ (total) and FIQ r=0.26, p < 0.05; PSQ-minor and FIQ r= 0.31,p < 0.01; PSQ-moderate and FIQ r= 0.19; PSQ (total) and CSI r=0.33, p < 0.01; PSQ- minor and CSI r= 0.36, p < 0.01; PSQ- moderate and CSI r=0.29, p < 0.05; PSQ (total) and CPT (pain intensity) r= 0.65, p = 0.01; PSQ (subscales) and CPT (pain intensity) r= 0.57- 0.60, P= 0.01; PSQ (total) and CPT (Tolerance) r= -0.56, p = 0.01; PSQ (subscales) and CPT (Tolerance) r= - 0.52 to -0.57, p = 0.01; PSQ (total) and PPT r= -0.59 p = 0.01; PSQ( subscales) and PPT r= -0.50 to -0.60, p = 0.01. (13+, 3-) |  | Fors et al 2020 (V) | Convergent validity→ FS (WPI+SSS) and FIQ rh= 0.74; WPI and FIQ rh= 0.59; SSS and FIQ rh= 0.85; FS (WPI+SSS) and TPC rh= 0.63; WPI and TPC rh= 0.55 SSS and TPC rh= 0.61 (6+) |  |
|  | Bakhtadze 2022 (I) | Convergent validity → CSI and NDI-RU rS=0.57, p<0.05; CSI and HADS ( anxiety, depression) rS=0.57- 0.56, p <0.05; CSI and McGill Pain Questionnaire (SF-MPQ-2) rS=0.46, p<0.05).(3+) |  | Inal 2021 (A) | Convergent validity→ PSQ (total) and BPI (pain, interference) r= 0.28-0.31, p < 0.05; PSQ (subscale)and BPI (pain) r= 0.24-0.31, p < 0.05; PSQ (subscale)and BPI (interference) r= 0.22-0.34, PSQ (total) and BDI r= 0.14, P>0.05; PSQ (subscale) and BDI r= 0.10-0.19, P>0.05; PSQ (total) and BAI r= -0.01, P>0.05; PSQ (subscale) and BAI r= -0.44 to 0.09, P>0.05; PSQ (total) and PCS r= 0.02, P>0.05; PSQ (subscale) and PCS r= - 0.02 to 0.12, P>0.05; PSQ (total) and VAS1 r= 0.70, P<0.001; PSQ (subscale) and VAS1 r= 0.63-0.68, P<0.001; PSQ (total) and VAS2 r= 0.82, P<0.001; PSQ (subscale) and VAS2 r= 0.76-0.79, P<0.001; (7+, 9-) |  | Jiao 2023 (V) | Convergent validity→ FS (WPI+SSS) and   FIQR rh= 0.487; WPI and FIQR rh= 0.292; SSS and FIQR rh= 0.589. (2+,1-) |  |

| **Table 6 COSMIN ratings on methodological quality, results, and overall rating per measurement property *cont.*** | | | | | | | | | |
| --- | --- | --- | --- | --- | --- | --- | --- | --- | --- |
| **COSMIN measurement properties** | **CSI [30-66]** | | | **PSQ 46,67-73]** | | | **FSQ [74-82]** | | |
|  | **Studies (Meth Qual Rating)** | **Results (Rating)** | **Summary of Results (Overall Rating)** | **Studies (Meth Qual Rating)** | **Results (Rating)** | **Summary of Results (Overall Rating)** | **Studies (Meth Qual Rating)** | **Results (Rating)** | **Summary of Results (Overall Rating)** |
|  | **V/A/D/I*** | **+/−/? **** | **+/−/±/?**** | **V/A/D/I*** | **+/−/? **** | **+/−/±/?**** | **V/A/D/I*** | **+/−/? **** | **+/−/±/?**** |
| **Hypothesis testing (construct validity)** | Wiangkham 2023 (V) | Convergent validity → CSI and VAS (pain intensity) rs= 0.36, P<0.001; CSI and NDI rs= 0.59 <0.001; CSI and FABQ (total score) rs= 0.42, <0.001; CSI and FABQ (Physical Activity and work) rs= 0.37-0.44, <0.001; CSI and PCS (total score) rs= 0.10, P= 0.07; CSI and PCS (subscale) rs= 0.10- 0.11, P= 0.06; CSI and SF-36 (total score) rs= –0.58, P<0.001; SF-36 (mental and physical) rs= –0.48 to- 0.54, P<0.001; (6+, 2-) |  | Latka 2019 (I) | Convergent validity→ PSQ and CSQ- total rho= 0.27, P<0.01. (1-) |  | Kang 2019 (A) | Convergent validity→ WPI and FIQR rh= 0.815; SSS and FIQR rh= 0.854;m WPI and EQ-5D rh= 0.717; SSS and EQ-5D rh= 0.724; WPI and MD-HAQ rh= 0.712; SSS and MD-HAQ rh=0.743. (6+) |  |
|  | Hendriks 2020 (A) | Convergent validity → CSI and SCL-90-R r_s_ =0.648, P <0.001, CSI and 4DSQ (somatization) r_p_= 0.681, P<0.001, CSI and 4DSQ (anxiety, depression, distress) r_s_= 0.54-0.66, P <0.001; CSI and IES r_s_= 0.450, P <0.001, CSI and TSK r_p_= 0.219, P=0.020; CSI and CIS20R r_s_ = 0.436, P<0.001; CSI and VAS r_s_ = 0.187, P=0.048; CSI and PPT (quadriceps, trapezius) rs= - 0.20 to –0.11; CSI and TS (quadriceps, trapezius) rp= –0.008 to 0.028, P >0.05. (5+, 4-) |  | Grundström 2019 (V) | Convergent validity→ PSQ ( total) and HPTm r= - 0.63, P<0.001; PSQ (total) and CPTm r= 0.56, P<0.001; PSQ (total) and PPTm r= - 0.43, P<0.001; PSQ ( total) and HADS r = 0.19-0.27, P<0.001; PSQ (subscale) and HPTm r= - 0.53 to -0.65, P<0.001; PSQ (moderate) and CPTm r= 0.44, p=0.006; PSQ (minor) and CPTm r= 0.60, P<0.001; PSQ (subscale) and PPTm r= - 0.34 to – 0.46, P<0.001; PSQ-moderate and HADS r = -0.03 to 0.23, P>0.05; PSQ-minor and HADS r = 0.31-0.38. (5+, 5-) |  | Moore 2022 (V) | Convergent validity→ FSQ and PPT rh = -0.13 to -0.20; FSQ and TS rh =0.12-0.13; No significant correlation was found between FSQ and CPM. The degree of correlation between QST and individual components of FSQ did not exhibit any substantial differences. (3-) |  |
|  | Bakhtadze 2021 (I) | Convergent validity → CSI and NDI-RU rs=0.56, p<0.05; CSI and ODI-RU (rS = 0.36, p <0.05). (2+) |  | Kim 2014 (A) | Convergent validity→ PSQ ( total) and PCS r = 0.38, P = 0.002; PSQ (subscale) and PCS r = 0.36-0.37, P = 0.003. (2+) |  | Aguirre Cárdenas 2021 (V) | Convergent validity→FSQ and PHQ-15(r = 0.62, p < 0.0001; FSQ and FIQ-R r = 0.60, p < 0.0001; FSQ and NPRS r = 0.51, p < 0.0001; FSQ and PHQ-9 r = 0.49, p < 0.0001; FSQ and BPI-PI r = 0.47, p < 0.0001; FSQ and PCS r = 0.31, p < 0.0001; FSQ and SF-12 r = −0.46, p < 0.0001; FSQ and PVAQ r = 0.22, p = 0.002. Discriminative validity → The FM group scored significantly higher on the FSQ total score than the RA group and G group, p < 0.0001. (8+, 1-) |  |

| **Table 6 COSMIN ratings on methodological quality, results, and overall rating per measurement property *cont.*** | | | | | | | | | | | |
| --- | --- | --- | --- | --- | --- | --- | --- | --- | --- | --- | --- |
| **COSMIN measurement properties** | **CSI [30-66]** | | | **PSQ 46,67-73]** | | | | **FSQ [74-82]** | | | |
|  | **Studies (Meth Qual Rating)** | **Results (Rating)** | **Summary of Results (Overall Rating)** | **Studies (Meth Qual Rating)** | **Results (Rating)** | **Summary of Results (Overall Rating)** | | **Studies (Meth Qual Rating)** | **Results (Rating)** | **Summary of Results (Overall Rating)** | |
|  | **V/A/D/I*** | **+/−/? **** | **+/−/±/?**** | **V/A/D/I*** | **+/−/? **** | **+/−/±/?**** | | **V/A/D/I*** | **+/−/? **** | **+/−/±/?**** | |
| **Hypothesis testing (construct validity)** | Sharma 2020 (A) | Convergent validity → CSI and PCS r = 0.50, P < 0.001; CSI and NRS pain intensity r = 0.25, P = 0.013; Discriminative validity → Mean CSI scores were significantly higher in women compared to men, P = 0.005. (2+, 1-) |  | Coronado and George, 2018 (A) | PSQ (total scores) and QST (pressure pain threshold, heat pain thresholds, suprathreshold heat pain rating) rho =−0.27 to 0.17; PSQ (minor) and QST (pressure pain threshold, heat pain thresholds, suprathreshold heat pain rating) rho = -0.18 to 0.12, p > 0.05; PSQ (total) and BRS rho = −0.39, p < 0.05; PSQ (minor) and BRS rho = −0.37, p < 0.05; PSQ (total) and DASS (anxiety) rho = 0.31, p < 0.05; PSQ(minor) and DASS (anxiety) rho = 0.25, p < 0.05; PSQ (total) and PANAS (negative) rho = 0.31, p < 0.05; PSQ(minor) and PANAS (negative) rho = 0.27, p <0.05. (4+, 4- ) | |  | Neville 2018 (V) | Convergent validity→ Female: FSQ and QST(PPT) = all \|r\| ≥ 0.27, all p ≤ 0.021 except thumbnail PPT; FSQ and TM r = 0.22, p = 0.078; FSQ and CPM r = 0.01, p= 0.945. No QST outcomes correlated with FM score in males. (6-) |  | |
|  | Düzce Keleş 2021 (V) | Discriminative validity → The fibromyalgia subgroup scored higher on the CSI score (≥ 10 points)than the CSP subgroup and the healthy control group scored lowest, p <0.001. (1+) |  |  |  |  | | Bidari 2015 (A) | Convergent validity→ FSQ (total) and FIQ (total) r= 0.45, P < 0.05; FSQ (subscale) and FIQ (total) r= 0.33- 0.49, P < 0.05; FSQ (total) and FIQ (function) r= 0.06, P > 0.05; FSQ (subscale) and FIQ (function) r= 0.05-0.10, P > 0.05; FSQ (total) and FIQ (symptom ) r= 0.24, P < 0.05; FSQ (subscale) and FIQ (symptom) r= 0.21-0.24, P < 0.05; FSQ (total) and SF-12 (PCS, MCS) r= -0.38 to -0.45, P < 0.05; FSQ (subscale) and SF-12 (PCS, MCS) r= -0.30 to -0.48, P < 0.05. Discriminative validity → The mean score of FSQ (PSD) and its components in the FM group were significantly higher than in non- FM chronic pain group, p=0.01. (5+, 4-) | |  |

| **Table 6 COSMIN ratings on methodological quality, results, and overall rating per measurement property *cont.*** | | | | | | | | | |
| --- | --- | --- | --- | --- | --- | --- | --- | --- | --- |
| **COSMIN measurement properties** | **CSI [30-66]** | | | **PSQ 46,67-73]** | | | **FSQ [74-82]** | | |
|  | **Studies (Meth Qual Rating)** | **Results (Rating)** | **Summary of Results (Overall Rating)** | **Studies (Meth Qual Rating)** | **Results (Rating)** | **Summary of Results (Overall Rating)** | **Studies (Meth Qual Rating)** | **Results (Rating)** | **Summary of Results (Overall Rating)** |
|  | **V/A/D/I*** | **+/−/? **** | **+/−/±/?**** | **V/A/D/I*** | **+/−/? **** | **+/−/±/?**** | **V/A/D/I*** | **+/−/? **** | **+/−/±/?**** |
| **Hypothesis testing (construct validity)** | Salaffi 2022 (V) | Convergent validity → CSI and modFAS ρ = 0.58 p<0.0001; CSI and FIQR *ρ* = 0.542, p<0.0001; CSI and PDS *ρ* = 0.518, p<0.0001 Discriminative validity → The CSI score among the FM very severe subgroup was higher than other severity subgroups and the remission subgroup scored lowest, p<0.0001. (4+) |  |  |  |  |  |  |  |
|  | Tanaka 2017 (A) | Convergent validity → CSI and EQ-5D r = −0.44, p<0.01; CSI and BPI (pain intensity) r = 0.42, p < 0.01; CSI and BPI (pain interference) r = 0.48, p < 0.01. Discriminative validity → one CSS group and 2 or more CSS groups scored higher on the CSI (≥10 points) than those with no CSS diagnosis group, p < 0.01. (4+) |  |  |  |  |  |  |  |
|  | Neblett 2013 (A) | Discriminative validity → Patients with diagnoses of CSS scored higher on the CSI (≥10 points) than the non-CSS patient sample and nonpatient control group, P =0 .05; with the AUC for the CSI=0 .86. (1+) |  |  |  |  |  |  |  |
|  | Caumo 2017 (A) | Convergent validity → CSI and PCS (subscale) r=0.62- 0.68; CSI and PCS (total score) r=0.68. Discriminative validity → Patients with diagnoses of FM were higher on the CSI (≥10 points) than other patient subgroups and the nonpatient control group, p<0.001. (3+) |  |  |  |  |  |  |  |
|  | Kregel 2016 (V) | Discriminative validity → The patient group scored higher on CSI than the control group. (1+) |  |  |  |  |  |  |  |
|  | Coronado and George 2018 (A) | Convergent validity → CSI and QST (pressure pain threshold, heat pain thresholds, suprathreshold heat pain rating) rho = −0.13 to 0.13, (p > 0.05); CSI and BRS rho = −0.29, p < 0.05; CSI and DASS-21 (depression, anxiety, and stress) rho = 0.64 - 0.67, p < 0.05) CSI and PANAS (negative) rho = 0.67, p < 0.05. (2+, 2-) |  |  |  |  |  |  |  |
|  | Feng 2022 (I) | Convergent validity → CSI and EQ-5D index r= −0.375, P <0.001; CSI and HADS (anxiety and depression) r= 0.467-0.525, P <0.001. Discriminative validity → The CSI scores in chronic pain patients with 2 or above CSSs were significantly higher (≥10 points), compared with those without CSS, p < 0.001. (3+) |  |  |  |  |  |  |  |
|  | Knezevic 2018 (V) | Discriminative validity → The patient subgroups scored significantly higher on the CSI total Scores (≥10 points) than the pain-free group, p < 0.001. (1+) |  |  |  |  |  |  |  |

| **Table 6 COSMIN ratings on methodological quality, results, and overall rating per measurement property *cont.*** | | | | | | | | | |
| --- | --- | --- | --- | --- | --- | --- | --- | --- | --- |
| **COSMIN measurement properties** | **CSI [30-66]** | | | **PSQ 46,67-73]** | | | **FSQ [74-82]** | | |
|  | **Studies (Meth Qual Rating)** | **Results (Rating)** | **Summary of Results (Overall Rating)** | **Studies (Meth Qual Rating)** | **Results (Rating)** | **Summary of Results (Overall Rating)** | **Studies (Meth Qual Rating)** | **Results (Rating)** | **Summary of Results (Overall Rating)** |
|  | **V/A/D/I*** | **+/−/? **** | **+/−/±/?**** | **V/A/D/I*** | **+/−/? **** | **+/−/±/?**** | **V/A/D/I*** | **+/−/? **** | **+/−/±/?**** |
| **Hypothesis testing (construct validity)** | Mikkonen 2021 (A) | Convergent validity → CSI and TSK (kinesiophobia) r = 0.463; CSI and RMDQ (disability) r = 0.387; CSI and the DEPS (depression) r = 0.615; CSI and PSQ-3 (impact of pain on sleep) r = 0.505; CSI and EQ-5D-5L (quality of life) r = −0.554. Discriminative validity →The CSI scores in patient subgroups were significantly higher (≥10 points) compared with those pain-free controls, < 0.001 (6+) |  |  |  |  |  |  |  |
|  | Neblett 2017 (V) | Discriminative validity→ The mean CSI scores moved up into higher severity ranges from the non-CSS patients to the patients with multiple CSSs. (1+) |  |  |  |  |  |  |  |
|  | Kosińska 2021 (V) | Convergent validity → CSI and NDI r=0.593; CSI and ODI r=0.422. Discriminative validity → CSI values were statistically higher (≥10 points) in the patient subgroup with both pain locations (CNP and CLBP) compared with only one location (CNP or CLBP), p<0.03. (3+) |  |  |  |  |  |  |  |
|  | Bilika 2020 (V) | Convergent validity → CSI and PCS r = 0.68. Discriminative validity → The FM group scored the highest on the total CSI scores (≥10 points) than other subgroups whereas the control group scored the lowest, p=0.000 (2+) |  |  |  |  |  |  |  |
|  | Kregel 2018 (V) | Convergent validity → CSI and pain intensity r = 0.320, P < 0.001; CSI and SF-36 (emotional, physical) r = -0.635 to -0.617, P < 0.001; CSI and PCS r = 0.464, P < 0.001; CSI and PDI r = 0.472, P < 0.001; CSI and PPT r = -0.276 to -0.237, P ≤ .01; CSI and CPM r = 0.017, P = 0.858. (4+, 2-) |  |  |  |  |  |  |  |
|  | Kim 2020 (V) | Convergent validity → CSI and WOMAC( pain scores) r = 0.524, p < 0.001; CSI and VAS r = 0.496, p < 0.001; CSI and WOMAC (function) r = 0.408, p < 0.001; CSI and EQ-5D r = 0.437, p < 0.001. (4+) |  |  |  |  |  |  |  |
|  | van der Noord 2018 (V) | Convergent validity → CSI and SCL-90 (anxiety, depressive) rs = 0.65- 0.67; p < .01; CSI and WPI rs = 0.43; p < .01; CSI and NRS rs = 0.36; p < .01; CSI and PCS rs = 0.39; p < .01. (5+) |  |  |  |  |  |  |  |
|  | Chiarotto 2018 (A) | Convergent validity → CSI-I score and NRS r= 0.427, P < 0.01; CSI-I score and SF36-PF score r= -0.479, P < 0.01; CSI-I score and HADS ( Anxiety) r= 0.706, P < 0.01; CSI-I score and HADS (depression) r= 0.551, P < 0.01; CSI-I score and PSEQ r= −0.618 P < 0.01; CSI-I score and ODI r= 0.356, P < 0.01; CSI-I score and RMDQ r= 0.450, P < 0.01. Discriminative validity → FM patients was ≥ 10 points higher than in the other subgroups, p < 0.001. (6+) |  |  |  |  |  |  |  |
|  | Valera-Calero 2022 (A) | Convergent validity → CSI and NRS r= 0.305, p<0.001; CSI and PPT (mastoid) r= −0.372, p<0.001; CSI and HADS (Depression, Anxiety) r= 0.415- 0.541, p<0.001. (3+) |  |  |  |  |  |  |  |

| **Table 6 COSMIN ratings on methodological quality, results, and overall rating per measurement property *cont.*** | | | | | | | | | |
| --- | --- | --- | --- | --- | --- | --- | --- | --- | --- |
| **COSMIN measurement properties** | **CSI [30-66]** | | | **PSQ 46,67-73]** | | | **FSQ [74-82]** | | |
|  | **Studies (Meth Qual Rating)** | **Results (Rating)** | **Summary of Results (Overall Rating)** | **Studies (Meth Qual Rating)** | **Results (Rating)** | **Summary of Results (Overall Rating)** | **Studies (Meth Qual Rating)** | **Results (Rating)** | **Summary of Results (Overall Rating)** |
|  | **V/A/D/I*** | **+/−/? **** | **+/−/±/?**** | **V/A/D/I*** | **+/−/? **** | **+/−/±/?**** | **V/A/D/I*** | **+/−/? **** | **+/−/±/?**** |
| **Hypothesis testing (construct validity)** | Madi 2021 (A) | Convergent validity → CSI and VRS rs= 0.38, p < 0.0001; CSI and PCS rs= 0.43, p < 0.0001; CSI and PCS (subscales) rs= 0.30- 0.44, p < 0.0001; CSI and EQ-VAS rs= -0.61, p < 0.0001; CSI and EQ-5D-3L rs= -0.48, p < 0.0001. Discriminative validity → Patients with more than one chronic pain complaint had significantly higher scores than patients with only one complaint, p= 0.002 and patients with a confirmed diagnosis of CS or CSS than those with no confirmed diagnosis, p = 0.04 (7+) |  |  |  |  |  |  |  |
|  | Knezevic 2020 (V) | Convergent validity → CSI and pain intensity τb = 0.271, P< 0.001; CSI and FACS (total) τb= 0.381, P< 0.001; CSI and FACS (Factor 1) τb = 0.410, P< 0.001; CSI and FACS (Factor 2) τb = 0.241 P< 0.001; CSI and PCS (total) τb= 0.369, P< 0.001; CSI and PCS (magnification, helplessness) τb= 0.343-0.400, P< 0.001; CSI and PCS (Rumination) τb= 0.290 P< 0.001; CSI and ODI τb= 0.381, P< 0.001; CSI and MOS (cognitive functioning scale, sleep scale) τb= -0.409 to -0.504, < 0.001; CSI and SF-36v2-( PCS) τb= -0.292, P< 0.001; CSI and SF-36v2-(MCS) τb= -0.402, P< 0.001; CSI and MSPSS (total) τb= -0.186, P= 0.043; CSI and MSPSS (subscales) τb= - 0.177 to -0.138. Discriminative validity → The CSI scores of the fibromyalgia group were significantly higher (≥10 points) than those of all other subgroups, while the control group exhibited lower scores than all patient groups, p < 0.001. (8+, 6-) |  |  |  |  |  |  |  |
|  | Van Wilgen 2018 (V) | Convergent validity → CSI and SCL-90 rs= 0.75, P < 0.001; CSI and WPI rs= 0.58, P < 0.001); CSI and VAS rs = 0.29, P < 0.01; CSI and PCS r = 0.27, P < 0.01. (2+, 2-) |  |  |  |  |  |  |  |
|  | Klute 2021 (A) | Convergent validity → CSI and PSQminor τ = 0.23; p < 0.001; CSI and PHQ15 τ = 0.57; p < 0.001; CSI and FSQ (SSS) τ = 0.56; p < 0.001; CSI and FSQ (WPI )τ = 0.47; p < 0.001; CSI and PainDETECT τ = 0.43; p < 0.001; CSI and MPSS τ = 0.32; p < 0.001; CSI and PCS τ = 0.28; p < 0.001 Discriminative validity → The CSI scores of the FMS group were significantly higher (≥10 points) than those of all other subgroups, while the control group exhibited lower scores than all patient groups, p < 0.001. (6+, 2-) |  |  |  |  |  |  |  |
|  | Holm 2021 (A) | Convergent validity → CSI and ODI rs = 0.52, p= <0.001; CSI and KEDS rs = 0.74, p= <0.001; CSI and WAI1 rs = -0.42, p= <0.001; CSI and QST (total) r = 0.22, p=0.008. (3+, 1-) |  |  |  |  |  |  |  |
|  | Neblett 2015 (A) | Discriminative validity → The CSS patient group had significantly higher total CSI scores (≥10 points) than the non-CSS patient group, < 0.001. (1+) |  |  |  |  |  |  |  |

| **Table 6 COSMIN ratings on methodological quality, results, and overall rating per measurement property *cont.*** | | | | | | | | | |
| --- | --- | --- | --- | --- | --- | --- | --- | --- | --- |
| **COSMIN measurement properties** | **CSI [30-66]** | | | **PSQ 46,67-73]** | | | **FSQ [74-82]** | | |
|  | **Studies (Meth Qual Rating)** | **Results (Rating)** | **Summary of Results (Overall Rating)** | **Studies (Meth Qual Rating)** | **Results (Rating)** | **Summary of Results (Overall Rating)** | **Studies (Meth Qual Rating)** | **Results (Rating)** | **Summary of Results (Overall Rating)** |
|  | **V/A/D/I*** | **+/−/? **** | **+/−/±/?**** | **V/A/D/I*** | **+/−/? **** | **+/−/±/?**** | **V/A/D/I*** | **+/−/? **** | **+/−/±/?**** |
| **Responsiveness** | Neblett 2017 (V) | There was a significant change in CSI scores (change score 10.2) between admission (without FRP-receiving patients) and discharge (with FRP-receiving patients) (p < 0.001). (+) | **Results in line with two hypothesis (+)** | NA | NA | NA | NA | NA | NA |
|  | Bid 2017 (V) | Significant changes were observed in CSI scores across three assessments for two groups of patients with chronic non-specific low back pain who underwent either a conventional physiotherapy program or a McKenzie exercise program (p < 0.001). The McKenzie program yielded significantly better results. (+) |  |  |  |  |  |  |  |

| **Table 6 COSMIN ratings on methodological quality, results, and overall rating per measurement property *cont.*** | | | | | | | | | |
| --- | --- | --- | --- | --- | --- | --- | --- | --- | --- |
| **COSMIN measurement properties** | **NFF [83]** | | | **GPQ [84]** | | | **SHS [85]** | | |
|  | **Studies (Meth Qual Rating)** | **Results (Rating)** | **Summary of Results (Overall Rating)** | **Studies (Meth Qual Rating)** | **Results (Rating)** | **Summary of Results (Overall Rating)** | **Studies (Meth Qual Rating)** | **Results (Rating)** | **Summary of Results (Overall Rating)** |
|  | **V/A/D/I*** | **+/−/? **** | **+/−/±/?**** | **V/A/D/I*** | **+/−/? **** | **+/−/±/?**** | **V/A/D/I*** | **+/−/? **** | **+/−/±/?**** |
| **Content validity** | N/A | N/A | N/A | N/A | N/A | N/A | N/A | N/A | N/A |
| **Structural validity** | N/A | N/A | N/A | van Bemmel 2019 (I) | Mokken analysis: no secondary dimensions emerged, item scalability > 0.30, (non-significant violations of monotonicity) and Adequate model fit. (+) | (+) | N/A | N/A | N/A |
| **Internal consistency** | Ghavidel-Parsa 2022 (V) | Cronbach’s α: s 0.72 but lack of redundancy between the items. (?) | (?) | van Bemmel 2019 (V) | Reliability coefficient r=0.90 (+) | (+) | Dixon 2016 (V) | Cronbach’s alpha: SHS (total)= 0.86, SHS (factors)= 0.62-0.88 (?) | (?) |
| **Cross-cultural validity** | N/A | N/A | N/A | N/A | N/A | N/A | N/A | N/A | N/A |
| **Reliability** | N/A | N/A | N/A | N/A | N/A | N/A | N/A | N/A | N/A |
| **Measurement error** | N/A | N/A | N/A | N/A | N/A | N/A | N/A | N/A | N/A |
| **Criterion validity** | Ghavidel-Parsa 2022 (V) | AUC= 0.87, sensitivity 82.5% and specificity 91.5% (+) | (+) | N/A | N/A | N/A | N/A | N/A | N/A |
| **Construct validity** | Ghavidel-Parsa 2022 (V) | Discriminative validity → The ROC AUC = 0.87 indicated a good ability of NFF to discriminate between FM and non-FM with a score of 4 as the best cut-off.(1+) | (+) | van Bemmel 2019 (A) | Convergent validity→ GPQ (total) and FSQ r= 0.72, <0.001; GPQ (total) and PDQ r= 0.87, <0.001; GPQ (total) and Pain intensity r= 0.81, <0.001; GPQ (total) and SF-36 (PCS) r= -0.62, <0.001; GPQ (total) and SF-36 (MCS) r= -0.75, <0.001; GPQ (total) and HAQ-DI r= 0.72, <0.001. Discriminative validity → Patients with FM scored significantly higher on the GPQ compared to patients with RA, P<0.001). The GPQ had excellent accuracy in predicting FM, with an AUC of 0.89. (6+) | (+) | Dixon 2016 (A) | Convergent validity→ SHS (total) and heat pain threshold r = -0.40, p = 0.019; SHS (total) and cold pressor duration r = -0.50, p = 0.002; SHS (total) and heat pain tolerance r= -0.009, p >0.05. Discriminative validity → study 4: SHS total scores of the fibromyalgia with osteoarthritis group were significantly higher (but ˂10 points) than those of the healthy control group, p˂ 0.001 Study 5: SHS total scores of the CLB patients did not significantly differ from the control group.   (2+,3-) | (-) |
| **Responsiveness** | N/A | N/A | N/A | N/A | N/A | N/A | N/A | N/A | N/A |

| **Table 6 COSMIN ratings on methodological quality, results, and overall rating per measurement property *cont.*** | | | | | | | |
| --- | --- | --- | --- | --- | --- | --- | --- |
| **COSMIN measurement properties** | **Novel self-report instrument [86]** | | | **L-VISS and VDS [87]** | | |  |
|  | **Studies (Meth Qual Rating)** | **Results (Rating)** | **Summary of Results (Overall Rating)** | **Studies (Meth Qual Rating)** | **Results (Rating)** | **Summary of Results (Overall Rating)** |  |
|  | **V/A/D/I*** | **+/−/? **** | **+/−/±/?**** | **V/A/D/I*** | **+/−/? **** | **+/−/±/?**** |  |
| **Content validity** | N/A | N/A | N/A | N/A | N/A | N/A |  |
| **Structural validity** | Austin 2020 (D) | EFA → factor 1 = 0.59- 0.91, factor 2= 0.60-0.91 (+) | **(+)** | NA | NA | NA |  |
| **Internal consistency** | Austin 2020 (V) | Cronbach’s α: factor 1 = 0.94, factor 2 = 0.90 (+) | **(+)** | Ten Brink 2021 (V) | Cronbach’s α: L-VISS = 0.85, VDS = 0.94 (?) | **(?)** |  |
| **Cross-cultural validity** | N/A | N/A | N/A | N/A | N/A | N/A |  |
| **Reliability** | N/A | N/A | N/A | N/A | N/A | N/A |  |
| **Measurement error** | N/A | N/A | N/A | N/A | N/A | N/A |  |
| **Criterion validity** | N/A | N/A | N/A | N/A | N/A | N/A |  |
| **Hypothesis testing (Construct validity)** | Austin 2020 (D) | Convergent validity→ Novel self-reported questionnaire (factor1, factor2) and PVAQ r= 0.54 – 0.58, p<0.0001; Novel self-reported questionnaire (factor1, factor2) and PASS-20 r= 0.65– 0.72, p<0.0001; Novel self-reported questionnaire (factor1, factor2) and PSEQ = -0.64 to – 0.71, p<0.0001; Novel self-reported questionnaire (factor1, factor2) and PCS r= 0.63 – 0.66, p<0.0001; Novel self-reported questionnaire (factor1, factor2) and DASS-21 Total r= 0.59 – 0.66, p<0.0001; Novel self-reported questionnaire (factor1, factor2) and CPM r= -0.41– 0.46, p=0.0001. (6+) | **(+)** | Ten Brink 2021 (D) | Convergent validity→ L-VISS and Visual Distortion Scores of the Pattern Glare Test (pattern 2) r= 0.33-0.42, p <.05; VDS and Visual Distortion Scores of the Pattern Glare Test (pattern 2) r=0.35-0.39, p <.05 for all patients except FM patient. Discriminative validity → Patients with fibromyalgia; Complex Regional Pain Syndrome (CRPS), and other types of pain exhibited elevated L-VISS and VDS scores compared to those without pain, p < .001. (3+ 1-) | **(+)** |  |
| **Responsiveness** | N/A | N/A | N/A | N/A | N/A | N/A |  |
| * V = very good, A = adequate, D = doubtful, I = inadequate; ** + = sufficient, - = insufficient, ± = inconsistent, ?= indeterminate; meth qual = methodological quality; CSI=Central sensitization Inventory; PSQ= Pain Sensitivity Questionnaire; FSQ= Fibromyalgia Survey Questionnaire; NFF= Nociplastic-based Fibromyalgia Feature; GPQ= Generalized Pain Questionnaire; SHS= Sensory Hypersensitivity Scale; L-VISS and VDS= Leiden Visual Sensitivity Scale and Visual Discomfort Scale; PROM= Patient-reported outcome measure; CFA = confirmatory factor analysis, CFI = comparative fit index, CTT = classical test theory, ICC = intraclass correlation coefficient, IRT = item response theory, MIC = minimal important change, RMSEA: root mean square error of approximation, SEM = standard error of measurement, SDC = smallest detectable change, SRMR: standardized root mean residuals, TLI = Tucker–Lewis index, QST=Quantitative sensory testing; BDI= Beck Depression Inventory; PCS= Pain Catastrophizing Scale; STAI= State-Trait Anxiety Inventory; PSD= polysymptomatic distress; PHQ-15 = Patient Health Questionnaire-15; FIQ= Fibromyalgia Impact Questionnaire; SF-12= Short‑Form‑12; HADS= Hospital Anxiety and Depression Scale; BPI= Brief Pain Inventory; RMQ= Roland-Morris Back Pain Questionnaire; VAS= Visual analogue scale; PHQ-4= Patient Health Questionnaire-4; CPT= Cold Pressor Test; PPT=Pressure pain threshold; TPC= tender points counts; NDI= Neck Disability Index; SF-MPQ-2= Short form McGill Pain Questionnaire-2; BAI= Beck Anxiety Inventory; FIQR= revised fibromyalgia impact questionnaire; FABQ= Fear- Avoidance Beliefs Questionnaire; SF-36= Short‑Form‑36; CSQ= Coping Strategy Questionnaire; EQ-5D= EuroQol five‐dimensional questionnaire; MD‐HAQ= Multidimensional Health Assessment Questionnaire; SCL-90-R= Symptom Checklist 90-R; 4DSQ= Four-Dimensional Symptom Questionnaire ; IES= Impact of Event Scale; TSK= Tampa Scale for Kinesiophobia; CIS20R= Checklist Individual Strength; TS= Temporal summation; HPT= Heat Pressor Test; ODI= Oswestry disability index; NPRS= Numeric Pain Rating Scale; PHQ-9= Patient Health Questionnaire-9; BPI-PI= Brief Pain Inventory–Interference Subscale; PVAQ= Pain Vigilance and Awareness Questionnaire; NRS=Numeric Rating Scale; BRS= Brief Resilience Scale; DASS= Depression, anxiety, and Stress Scale; PANAS= Positive and Negative Affect Schedule; CPM= conditioned pain modulation; modFAS= Modified Fibromyalgia Assessment Status; PDS= Polysymptomatic Distress Scale; RMDQ= Roland Morris Disability Questionnaire; PSQ-3= Pain and Sleep Questionnaire Three-Item Index; EQ-5D-5L= EuroQol five‐dimensional five-level questionnaire; PDI= Pain Disability Index; WOMAC= Western Ontario and McMaster Universities Osteoarthritis Index; PSEQ = Pain Self-Efficacy Questionnaire; EQ-5D-3L= EuroQol five‐dimensional three-level questionnaire; FACS= Fear-Avoidance Components Scale; MOS= Medical Outcomes Study; MSPSS= Multidimensional Scale of Perceived Social Support; MPSS= Mainz pain staging system; KEDS= Karolinska exhaustion disorder scale; WAI1= Work ability index item 1. AUC= Area under the curve. | | | | | | |  |
